# Molecular and Cellular Mechanisms of Teneurin Signaling in Synaptic Partner Matching

**DOI:** 10.1016/j.cell.2024.06.022

**Published:** 2024-07-11

**Authors:** Chuanyun Xu, Zhuoran Li, Cheng Lyu, Yixin Hu, Colleen N. McLaughlin, Kenneth Kin Lam Wong, Qijing Xie, David J. Luginbuhl, Hongjie Li, Namrata D. Udeshi, Tanya Svinkina, D. R. Mani, Shuo Han, Tongchao Li, Yang Li, Ricardo Guajardo, Alice Y. Ting, Steven A. Carr, Jiefu Li, Liqun Luo

**Affiliations:** 1 Department of Biology and Howard Hughes Medical Institute, Stanford University, Stanford, CA 94305, USA; 2 Biology Graduate Program, Stanford University, CA 94305, USA; 3 Neurosciences Graduate Program, Stanford University, CA 94305, USA; 4 The Broad Institute of MIT and Harvard, Cambridge, MA 02142, USA; 5 Departments of Genetics, Biology, and Chemistry, Chan Zuckerberg Biohub, Stanford University, Stanford, CA 94305, USA; 6 Janelia Research Campus, Howard Hughes Medical Institute, Ashburn, VA 20147, USA; 7 Present address: Huffington Center on Aging, Department of Molecular and Human Genetics, Baylor College of Medicine, Houston, TX 77030, USA; 8 Present address: Key Laboratory of RNA Science and Engineering, Center for Excellence in Molecular Cell Science, Shanghai Institute of Biochemistry and Cell Biology, Chinese Academy of Sciences, Shanghai, 200031, China; 9 Present address: Liangzhu Laboratory, MOE Frontier Science Center for Brain Science and Brain-machine Integration, State Key Laboratory of Brain-machine Intelligence, Zhejiang University, Hangzhou 311121, China; 10 Lead contact

## Abstract

In developing brains, axons exhibit remarkable precision in selecting synaptic partners among many non-partner cells. Evolutionally conserved teneurins are transmembrane proteins that instruct synaptic partner matching. However, how intracellular signaling pathways execute teneurin’s functions is unclear. Here, we use *in situ* proximity labeling to obtain the intracellular interactome of teneurin (Ten-m) in the *Drosophila* brain. Genetic interaction studies using quantitative partner matching assays in both olfactory receptor neurons (ORNs) and projection neurons (PNs) reveal a common pathway: Ten-m binds to and negatively regulates a RhoGAP, thus activating the Rac1 small GTPases to promote synaptic partner matching. Developmental analyses with single-axon resolution identify the cellular mechanism of synaptic partner matching: Ten-m signaling promotes local F-actin levels and stabilizes ORN axon branches that contact partner PN dendrites. Combining spatial proteomics and high-resolution phenotypic analyses, this study advanced our understanding of both cellular and molecular mechanisms of synaptic partner matching.

## In Brief

Synaptic partner matching in the fly olfactory circuit is achieved by selectively stabilizing axon branches by partner dendrites. Synaptic partner matching molecule Ten-m regulates this process by binding to and negatively regulating a RhoGAP, which in turn activates the Rac1 small GTPase to promote actin polymerization.

## Graphical Abstract

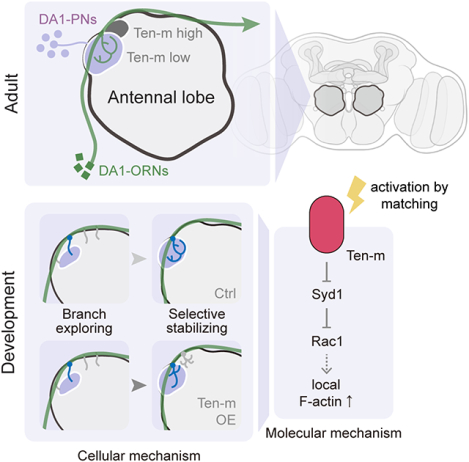

## INTRODUCTION

The precise assembly of neural circuits involves multiple developmental processes. Compared to axon guidance and dendrite morphogenesis, much less is known about cellular and molecular mechanisms that mediate synaptic partner matching^1–4^. Evolutionarily conserved teneurins are transmembrane proteins that instruct synaptic partner matching^5,6^. Teneurins also regulate diverse biological processes including cell polarity, neuronal migration, axon guidance and target selection, myelination, and synapse development^6–17^. The four human teneurins have been implicated in diseases including sensory and motor dysfunctions, neurodevelopmental and psychiatric disorders, and cancers^18–26^.

Teneurins are type II transmembrane proteins comprising a small intracellular amino terminus, a single transmembrane domain, and a large extracellular carboxyl terminus with evolutionarily conserved domains for protein-protein interactions^27^. Previous structural and functional studies of teneurins have largely focused on the cell-cell interactions mediated by their extracellular domains. These include EGF-like repeats essential for teneurin *cis*-dimerization, a beta-propeller region implicated in *trans*-homophilic binding, and a tyrosine- and glutamate-rich domain for heterophilic interactions with latrophilins, members of adhesion G-protein-coupled receptor family^9,28–36^. For example, homophilic attractions between mouse teneurin-3 regulate topographic target selection of hippocampal axons^13^, whereas heterophilic interactions between teneurin-3 and latrophilin-2 mediate reciprocal repulsions between axons and target neurons that express them^14,37^. Heterophilic interactions between teneurins and latrophilins also regulate neuronal migration^9^ and synapse formation in specific subcellular compartments^15^. Compared to the rich knowledge of the extracellular domains, little is known how intracellular signaling works to execute the diverse functions of teneurins. Indeed, it is unknown whether intracellular domains are required for any of teneurins’ functions.

**Figure 1.**
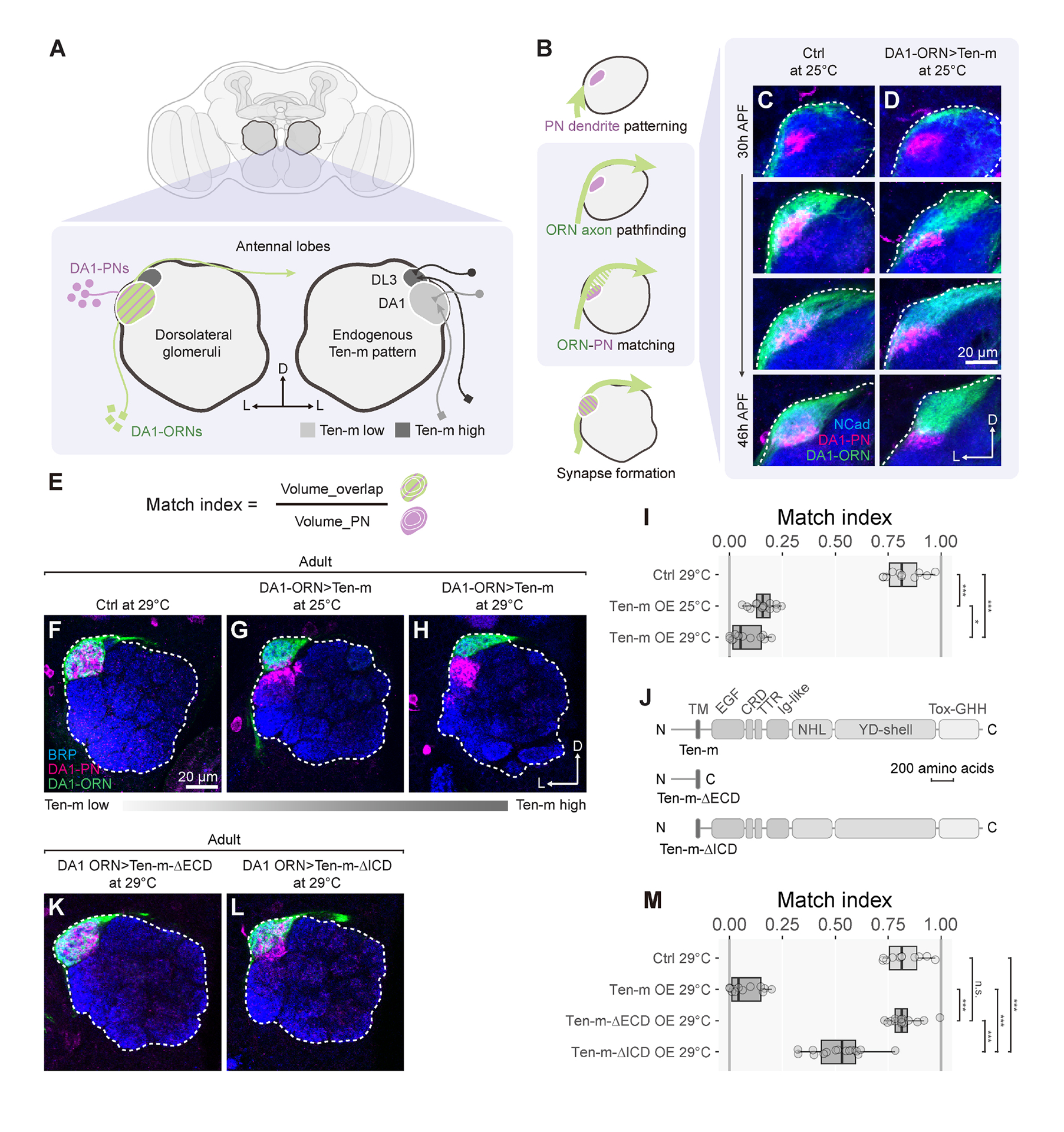
A quantitative gain-of-function assay for synaptic partner matching (A) Adult *Drosophila* brain schematic highlighting antennal lobes and locations of the DA1 and DL3 glomeruli. Left, DA1-ORN axons (green) synapse with DA1-PN dendrites (purple, contralateral projection omitted). Right, endogenous Ten-m levels are low in DA1-ORNs and DA1-PNs, but high in DL3-ORNs and DA1-PNs. (B) Schematic of sequential developmental steps of DA1 ORN-PN pairing. (C) Time course of control DA1-ORN axons (green, labeled by a membrane marker mCD8-GFP driven by *DA1*-*ORN*-*GAL4*, a split GAL4) innervating, elaborating, and coalescing with DA1-PN dendrites (magenta, labeled by a membrane-tagged tdTomato, driven by *Mz19*-*QF2)*. APF, after puparium formation. (D) Ten-m overexpression causes DA1-ORNs to elaborate more dorsomedially, resulting in only partial overlap between DA1-ORN axons and DA1-PN dendrites. (E) “Match index” definition. (F–H) Confocal sections of adult antennal lobes showing DA1-ORN axons (green) of control (F), Ten-m-overexpression at 25°C (G) and 29°C (H), and DA1-PN dendrites (magenta). (I) Match indices for (F–H). (J) Domain organization of Ten-m, Ten-m-ΔECD, and Ten-m-ΔICD. TM, transmembrane domain; see Methods for domain abbreviations. (K, L) Confocal sections of adult antennal lobes showing DA1-ORN axons (green) overexpressing Ten-m-ΔECD (K) or Ten-m-ΔICD (L) at 29°C, and DA1-PN dendrites (magenta). (M) Match indices for (K) and (L). In this and all subsequent figures: D, dorsal; L, lateral. Dashed outlines, antennal lobe. NCad (N-cadherin) and BRP (Bruchpilot) are general neuropil markers. * p < 0.05; ** p < 0.01; *** p < 0.001; n.s., not significant. One-way ANOVA (with Tukey’s test) was used in (I) and (M). See Figures S1, S2 for additional data.

In the *Drosophila* olfactory circuit, ~50 types of olfactory receptor neurons (ORNs) synapse with 50 types of second-order projection neurons (PNs) to form precise 1-to-1 matching at 50 discrete glomeruli (Figure 1A), providing an excellent model for investigating mechanisms of synaptic partner matching. We previously found that two *Drosophila* teneurins, Ten-m (tenascin-major) and Ten-a (tenascin-accessory), are expressed in select matching ORN-PN pairs and instruct synaptic partner matching through homophilic attraction^5^. Here, we combine spatial proteomics and *in vivo* genetic interaction assays to investigate the intracellular signaling mechanisms that mediate this attraction. We find that Ten-m signals through a RhoGAP and the Rac1 small GTPase to regulate the actin cytoskeleton. Developmental analyses with single-axon resolution further reveal that this signaling pathway acts to selectively stabilize ORN axon branches that contact partner PN dendrites.

## RESULTS

### A quantitative gain-of-function assay for Ten-m signaling *in vivo*

To investigate Ten-m signaling mechanisms, we first sought to establish a quantitative assay in which altering Ten-m activity would lead to a robust phenotype *in vivo*. We can then examine how perturbing Ten-m’s signaling partner(s) would modify such a phenotype. We focused on DA1-ORNs that target their axons to the DA1 glomerulus and synapse with DA1-PN dendrites (Figure 1A). Both DA1-ORNs and DA1-PNs express Ten-m at low levels^5^. Utilizing orthogonal drivers and reporters, we simultaneously tracked DA1-ORN axons and DA1-PN dendrites across development in the same control (Figure 1C) or Ten-m-overexpressing (Figure 1D) animals.

During fly olfactory circuit assembly, PNs first pattern the antennal lobe by targeting dendrites to antennal lobe regions approximating their eventual glomerular positions (Figure 1B)^38,39^. At 30 hours after puparium formation (h APF), DA1-ORN axons extended along the antennal lobe surface without forming extensive contact with DA1-PN dendrites in both control and Ten-m-overexpression conditions. During the next 16 hours, control DA1-ORN axons initially elaborated over a larger region than DA1-PN dendrites and gradually coalesced axons with DA1-PN dendrites (Figure 1C). However, Ten-m-overexpressing DA1-ORN axons elaborated over a region more dorsomedial than the DA1-PN dendritic region, resulting in only partial overlap between DA1-ORN axons and DA1-PN dendrites throughout development (Figure 1D).

Quantification of the mismatching phenotype using a “match index” in the adult antennal lobe (Figure 1E) revealed substantial difference in control and Ten-m overexpression conditions (Figure 1F, H, I). To determine whether this mismatching phenotype depends on Ten-m overexpression levels, we exploited the temperature dependence of GAL4-driven transgene expression^40,41^ (Figure S1A–C, S1F), and observed a more pronounced Ten-m-overexpression phenotype at 29°C than at 25°C (Figure 1G–I). Thus, the match index provides an assay sensitive to Ten-m overexpression levels.

*Trans*-synaptic labeling^42^ revealed that mistargeted DA1-ORN axons likely matched with dendrites of DL3-PNs based on the location of the trans-synaptically labeled PN dendrites and terminal branching patterns of axons (Figure S2). These results underscore the like-to-like matching in teneurin levels between synaptic partners, as DL3-PNs express high levels of both Ten-m and Ten-a, paralleling Ten-m-overexpressing DA1-ORNs that normally express high levels of Ten-a^5^. Moreover, co-overexpressing Ten-m in DA1-PNs partially suppressed the mismatching phenotypes caused by overexpressing Ten-m in DA1-ORNs (Figure S1G, H). Thus, these gain-of-function phenotypes likely result from homophilic attraction between Ten-m-expressing ORNs and PNs.

### Both the extracellular and intracellular domains of Ten-m are required for signaling

Using our quantitative assay, we assessed the role of Ten-m’s extracellular and intracellular domains in mediating signaling by overexpressing *Ten*-*m* transgenes lacking the extracellular domain (ΔECD) or intracellular domain (ΔICD) (Figure 1J). All *Ten*-*m* transgenes were integrated into the same genomic locus, expressed proteins at a similar level *in vivo* (Figure S1D–F), and were trafficked to the cell surface (Figure S1I, J). Ten-m-ΔECD overexpression did not cause any mismatching phenotype (Figure 1K, M), while Ten-m-ΔICD overexpression caused a partial mismatching phenotype (Figure 1L, M). These experiments indicate that ECD is essential for mediating Ten-m’s gain-of-function effect. Signaling through ICD is also required for the full activity of Ten-m; the remaining mismatching phenotypes in Ten-m-ΔICD overexpression could be caused by homophilic adhesion between DA1-ORNs and non-partner PNs without intracellular signaling, or by a potential co-receptor of Ten-m that can mediate some intracellular signaling. Regardless, the substantial difference in the match index between overexpressing wild-type-Ten-m and Ten-m-ΔICD offers a quantitative assay for examining the Ten-m-ICD-dependent signaling mechanism.

### Proximity labeling to identify Ten-m-ICD interacting proteins *in situ*

**Figure 2.**
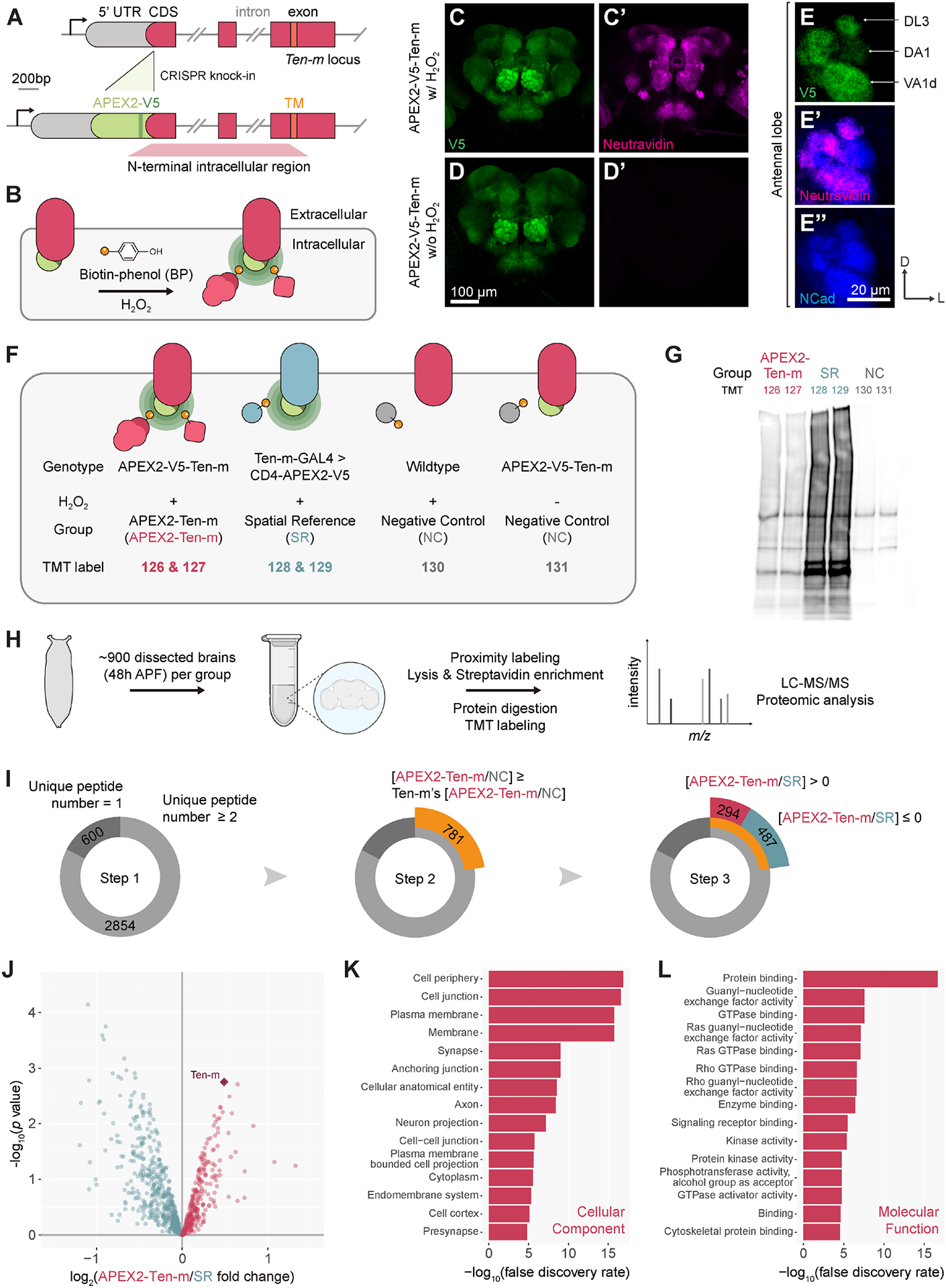
*In situ* spatial proteomics to identify proteins in physical proximity to Ten-m-ICD (A) CRISPR-knockin at the *Ten*-*m* gene locus. APEX2-V5 is N-terminal to the *Ten*-*m* coding sequence (CDS). TM, transmembrane domain. (B) Schematic of APEX2-based *in situ* proximity labeling for profiling the Ten-m intracellular interactome. (C and C’) V5 and Neutravidin staining of APEX2-V5-Ten-m fly brain after proximity labeling. (D and D’) Same as C and C’ without H2O2. (E–E”) Representative confocal images of an antennal lobe showing that Ten-m expression and APEX2 activity are high in the DL3, VA1d, and VA1v glomeruli but low in the DA1 glomerulus. (F) Design of the quantitative proteomic experiment. TMT labels indicate the TMT tags (e.g., 126) used in all groups. The APEX2-Ten-m and SR groups each contains two replicates. (G) Streptavidin blot of the post-enrichment bead elute. (H) Workflow of the Ten-m intracellular interactome profiling. (I) Numbers of proteins after each step of the ratiometric and cutoff analysis. (J) Volcano plot showing all proteins at step 3. Each dot represents a protein; Diamond, Ten-m. Proteins in red constitute the Ten-m intracellular interactome. (K, L) Top 15 Gene Ontology terms for cellular component (K) or molecular function (L) in the Ten-m intracellular interactome. See Figure S3 and Tables S1, S2 for additional data.

To investigate the molecular mechanisms by which Ten-m-ICD transduces signals, we next used proximity labeling^43–45^ to identify proteins in physical proximity to Ten-m-ICD in native tissues. Given the critical role of teneurin levels in synaptic matching, we used CRISPR-knockin to maintain endogenous Ten-m levels. We inserted the coding sequence of *APEX2*-*V5* N-terminal to the *Ten*-*m* coding sequence (Figure 2A) such that APEX2 would catalyze biotinylation of proteins in physical proximity to Ten-m-ICD in the presence of biotin-phenol and H_2_O_2_ (Figure 2B). Flies homozygous for the insertion allele were viable, whereas flies homozygous for *Ten*-*m* mutant are embryonic lethal^11^, suggesting that APEX2-V5 insertion did not disrupt native Ten-m function. APEX2-V5-Ten-m recapitulated endogenous Ten-m’s expression patterns^5^ (Figure 2C, E, E”). In the presence of biotin-phenol and H_2_O_2_, APEX2-V5-Ten-m catalyzed biotinylation with a similar spatial pattern as V5 staining (Figure 2C, C’, E, E’). No biotinylation was observed when H_2_O_2_ was omitted (Figure 2D, D’).

We next carried out large-scale proximity labeling experiments from pupal brains followed by quantitative mass spectrometry to identify Ten-m-ICD-interacting proteins during development. We devised a 6-plex tandem-mass-tag (TMT) design for ratiometric analysis, featuring an APEX2-V5-Ten-m group (to capture Ten-m-ICD interactors), a spatial reference (SR) group (to identify the background from generic proteins close to the plasma membrane), and a negative control (NC) group (omitting either H_2_O_2_ or APEX2 transgene to account for endogenously biotinylated and endogenous peroxidase-labeled proteins) (Figure 2F). For the SR group, CD4-APEX2-V5—a generic transmembrane protein with APEX2 at its intracellular C-terminus—was expressed in Ten-m-expressing cells (Figure 2F). Biochemical characterization of the post-enrichment eluate via streptavidin blot analysis revealed that both the APEX2-Ten-m and SR groups had much more biotinylated proteins, each with a distinct pattern, than the negative control group, indicating group-specific protein enrichment (Figure 2G). We dissected ~900 brains at 48h APF per TMT plex and processed the samples following previous protocols^46,47^ (Methods). After 6-plex TMT labeling, we pooled all samples for liquid chromatography-tandem mass spectrometry (LC-MS/MS) analysis (Figure 2H). Proteomes exhibited strong correlations between biological replicates (Figure S3A), suggesting high sample quality.

To identify Ten-m’s prospective interacting partners, we applied 3 steps in proteomic analysis. (1) We filtered a total of 3454 detected proteins from 6 samples, focusing on those with two or more unique peptides, resulting in 2854 proteins (Figure 2I, Step 1; Table S1). (2) To remove endogenously biotinylated and endogenous peroxidase-labeled proteins (NC lanes in Figure 2G), we used [APEX2-Ten-m/NC] fold change of the Ten-m protein itself (Figure 2F) as a cutoff and obtained 781 proteins (Figure 2I, Step 2; Table S1). (3) To remove generic proteins close to the cell membrane, we applied a [APEX2-Ten-m/SR] fold change-based ratiometric strategy (Figure 2F) and acquired 294 proteins enriched by APEX2-Ten-m (Figure 2I, Step 3; Figure 2J, red; Table S1)—hereafter, the Ten-m intracellular interactome.

Gene Ontology analysis indicated that the Ten-m intracellular interactome comprised proteins localized at the cell surface, synapse, cytoplasm, and endomembrane systems (Figure 2K). These proteins functionally relate to GTPase signaling pathways, kinase activity, signaling receptor binding, and cytoskeletal protein binding (Figure 2L, Figure S3B–D), reminiscent of partners of previously identified axon guidance receptors such as Eph receptors^48,49^ and Plexins^50–52^.

### Ten-m binds to and genetically interacts with Syd1, a GAP for Rho GTPases

**Figure 3.**
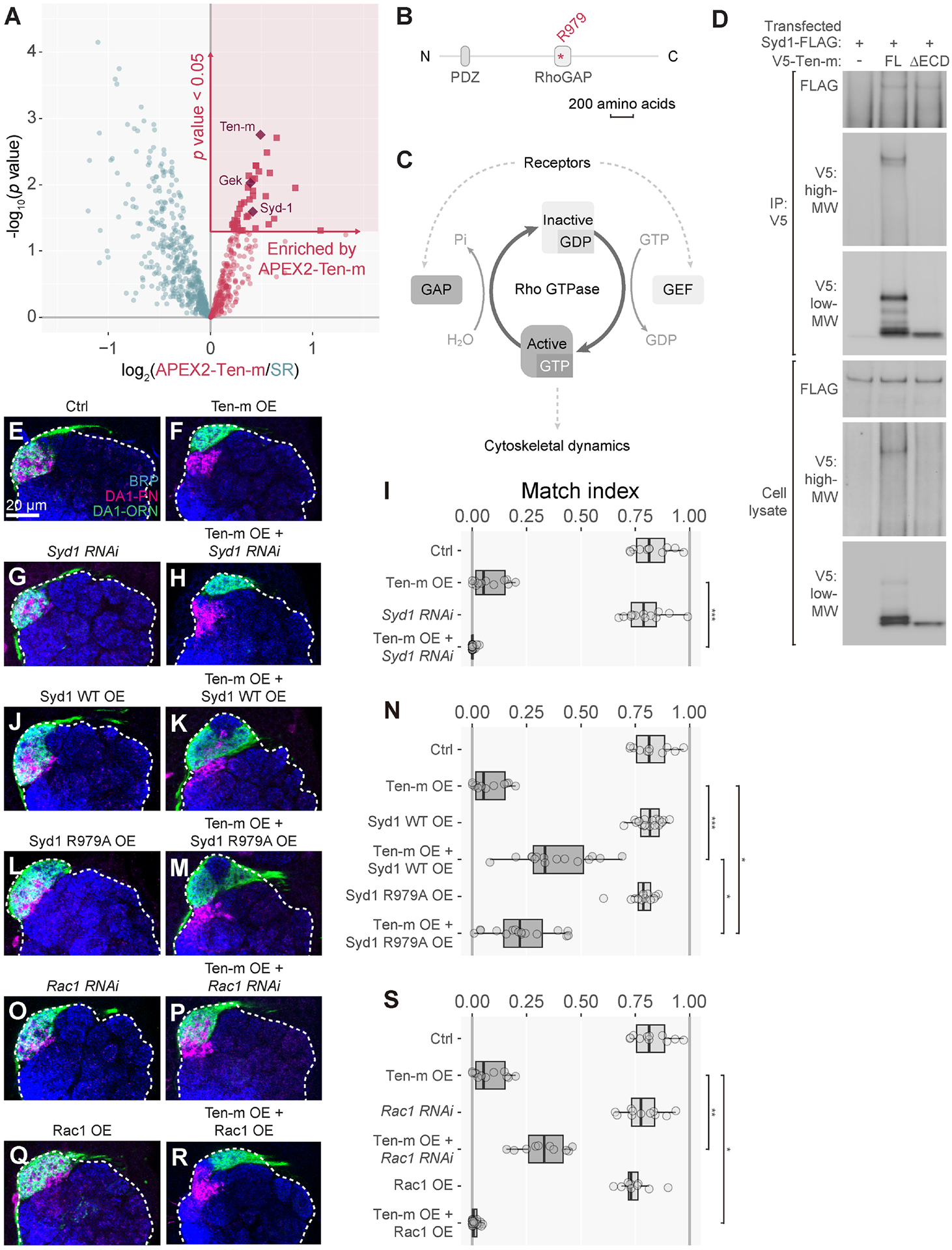
Ten-m interacts with Syd1 RhoGAP and Rac1 GTPase in ORNs (A) Selection criteria for the top candidate Ten-m-interacting proteins. Ten-m, Syd1, and Gek are highlighted with diamonds. (B) Domain organization of Syd1. *, a critical amino acid for RhoGAP activity. (C) The Rho GTPase cycle. Plasma membrane receptors control Rho GTPases through regulating guanine-nucleotide exchange factors (GEFs) or GTPase-activating proteins (GAPs), which switch Rho GTPases on or off, respectively. When GTP-bound, Rho GTPases bind to and activate effectors to regulate cytoskeletal dynamics. (D) Co-immunoprecipitation of V5-tagged Ten-m and FLAG-tagged Syd1 proteins from co-transfected S2 cells. MW, molecular weight. The low MW plot shows Ten-m-ΔECD (right) or proteolytic products of full-length (FL) Ten-m (middle). (E–I) Representative confocal images of DA1-PN dendrites (magenta) and DA1-ORN axons (green) of control (E), Ten-m overexpression (F), *Syd1*-*RNAi* alone (G), and Ten-m overexpression with *Syd1*-*RNAi* (H). Match indices in (I). (J–N) Representative confocal images of DA1-PN dendrites (magenta) and DA1-ORN axons (green) of Syd1 overexpression (J), Syd1 and Ten-m co-overexpression (K), Syd1-R979A overexpression (L), and Syd1-R979A and Ten-m co-overexpression (M). Match indices in (N). (O–S) Representative confocal images of DA1-PN dendrites (magenta) and DA1-ORN axons (green) of *Rac1*-*RNAi* (O), Ten-m overexpression with *Rac1*-*RNAi* (P), Rac1 overexpression (Q), and Ten-m and Rac1 co-overexpression (R). Match indices in (S). Kruskal-Wallis test with Bonferroni post-hoc correction for multiple comparisons was used in (I), (N), and (S). See Figure S4 for additional data.

Among the 37 proteins significantly enriched in the APEX2-Ten-m group relative to the spatial reference group (Figure 3A; Table S2) was RhoGAP100F (Syd1), the *Drosophila* homolog of *C. elegans* Syd-1, which functions in presynaptic assembly in worms and flies^53–55^. Syd1 has a GTPase-activating protein (GAP) domain for the Rho family of small GTPases (Figure 3B) and exhibits GAP activity towards Rac1 and Cdc42^56^. Given the central role for Rho GTPases in transducing extracellular signals to the cytoskeleton^57^ (Figure 3C), we next investigated the interactions between Ten-m and Syd1.

To test whether Ten-m physically interacts with Syd1, we expressed recombinant V5-tagged full-length Ten-m and FLAG-tagged full-length Syd1 in *Drosophila* S2 cells. Immunoprecipitation with a V5 antibody co-precipitated Syd1-FLAG (Figure 3D), indicating that Ten-m and Syd1 directly interact or belong to a same protein complex. Syd1-FLAG was also co-immunoprecipitated by Ten-m-ΔECD (Figure 3D), suggesting that Ten-m-ICD is sufficient to mediate interaction with Syd1.

To test whether Ten-m genetically interacts with Syd1, we examined whether knocking down or overexpressing Syd1 in DA1-ORNs would modify the Ten-m-overexpression phenotypes (Figure 1). Compared with Ten-m overexpression alone (Figure 3F), co-expressing *Syd1*-*RNAi* to knock down Syd1 in DA1-ORNs enhanced the mismatching phenotypes (Figure 3H, I). Syd1 knockdown alone did not affect the match index (Figure 3E, G, I). Conversely, co-expressing wild-type Syd1 in DA1-ORNs partially suppressed the mismatching phenotype of Ten-m overexpression (Figure 3K), while overexpressing Syd1 alone did not affect the match index (Figure 3J, N). We note that overexpressing Syd1 (alone or co-expressed with Ten-m) expanded the volume occupied by DA1-ORN axons, which may result from Syd1’s role in promoting presynaptic terminal development^54,56^.

We also tested the effect of overexpressing Syd1 with a point mutation (R979A) that abolishes its RhoGAP activity^56^. Syd1-R979A overexpression also caused DA1-ORN axon expansion (Figure 3L, M), suggesting that this activity does not depend on RhoGAP activity^56^. However, the suppression of ORN-PN mismatch was significantly reduced compared to expressing wild-type Syd1 (Figure 3M, N), suggesting that the regulation of synaptic partner matching by Syd1 is partially dependent on its RhoGAP activity.

Syd1 knockdown did not enhance mismatching phenotypes caused by overexpressing Ten-m-ΔICD (Figure S4A, B), suggesting that the residual function of Ten-m-ΔICD does not involve Syd1.

In summary, our data indicate that Syd1 physically and genetically interacts with Ten-m. The genetic experiments further suggest a negative interaction between Ten-m and Syd1 in target selection: increasing Syd1 levels decreases Ten-m signaling, whereas decreasing Syd1 levels increases Ten-m signaling.

### Ten-m genetically interacts with Rac1 GTPase

Given the reported RhoGAP activity of Syd1 towards Cdc42 and Rac1^56^, we next examined genetic interactions between Ten-m and Rho1, Cdc42, and Rac1 using the Ten-m-overexpression assay. Rho1 or Cdc42 knockdown in DA1-ORNs did not significantly affect the Ten-m-overexpression phenotype (Figure S4C–G). However, the Ten-m-overexpression phenotype was suppressed by Rac1 knockdown (Figure 3P, S) and enhanced by Rac1 overexpression (Figure 3R, S). Rac1 knockdown or overexpression alone did not significantly affect the match index (Figure 3O, Q, S).

**Figure 4.**
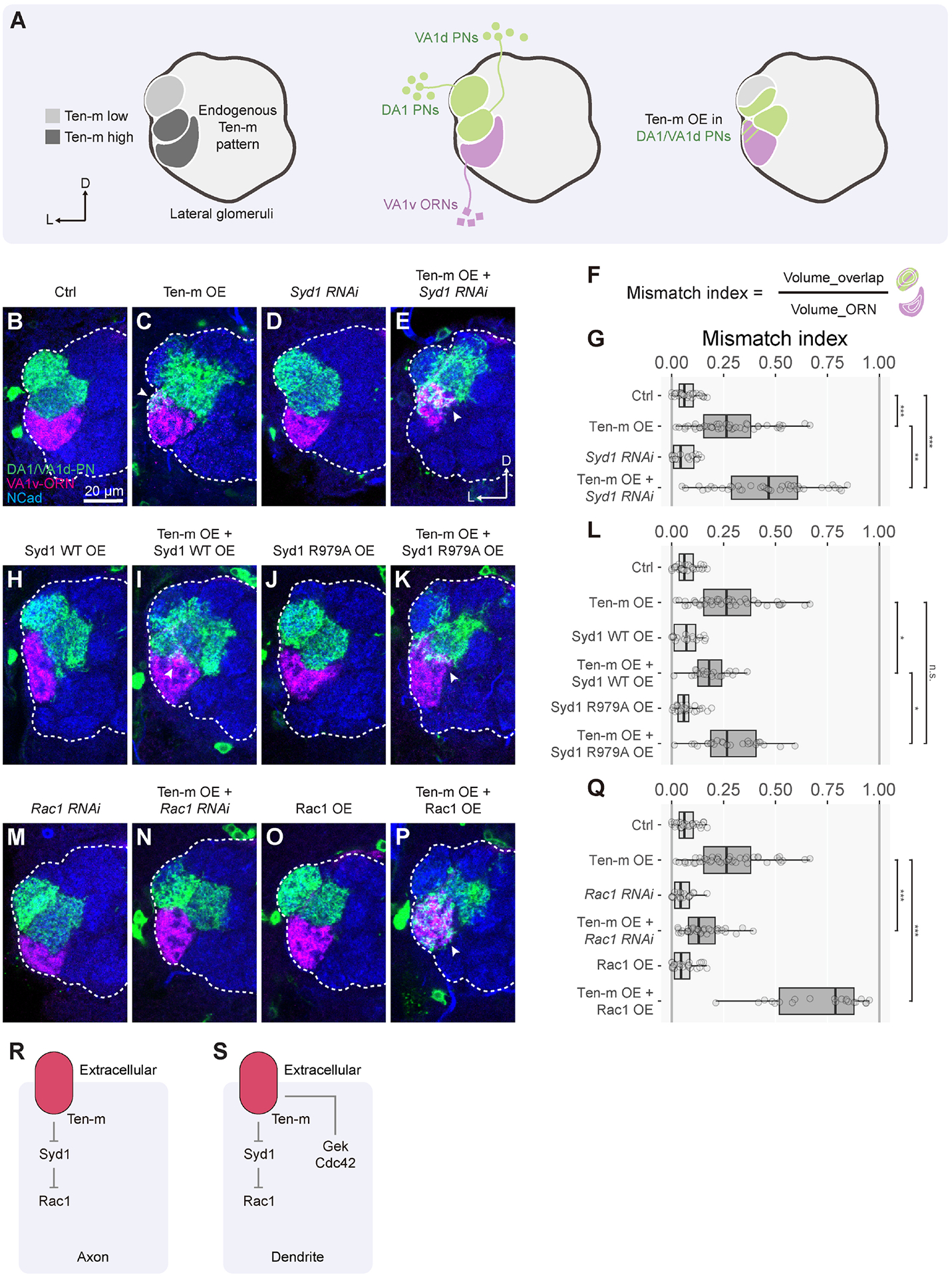
Ten-m interacts with Syd1 and Rac1 in PNs (A) Genetic interaction assay for Ten-m signaling in PN dendrites. Left, endogenous Ten-m levels. *Mz19*-*GAL4* is expressed in VA1d-PNs and DA1-PNs (green), whose dendrites do not overlap with VA1v-ORN axons (purple) in control (middle) but overlap with VA1v-ORN axons when overexpressing Ten-m (right). (B–E) Representative confocal images of VA1v-ORN axons (magenta) and Mz19-PN dendrites (green) of control (B), Ten-m overexpression (C), *Syd1*-*RNAi* (D), and Ten-m overexpression with *Syd1*-*RNAi* (E). (F, G) Mismatch index (F) for experiments in Panels B–E (G). (H–L) Representative confocal images of VA1v-ORN axons (magenta) and Mz19-PN dendrites (green) of Syd1 overexpression (H), Syd1 and Ten-m co-overexpression (I), Syd1-R979A overexpression (J), and Syd1-R979A and Ten-m co-overexpression (K). Quantified in (L). (M–Q) Representative confocal images of VA1v-ORN axons (magenta) and Mz19-PN dendrites (green) of *Rac1*-*RNAi* (M), Ten-m overexpression with *Rac1*-*RNAi* (N), Rac1 overexpression (O), and Ten-m and Rac1 co-overexpression (P). Quantified in (Q). (R, S) Summary and working models for Ten-m signaling in ORN axons (R) and PN dendrites (S). In both cases, Ten-m negatively regulates Syd1, and in turn activates Rac1 GTPase. Ten-m exhibits negative genetic interactions with Gek and Cdc42 only in PN dendrites. Arrowheads indicate overlap regions between Mz19-PNs and VA1v-ORNs. Kruskal-Wallis test with Bonferroni post-hoc correction for multiple comparisons was used in (G), (L), and (Q). See Figures S4, S5 for additional data.

Thus, Rac1 exhibited a positive genetic interaction with Ten-m. This is consistent with the negative genetic interaction between Syd1 and Ten-m—as a RhoGAP, Syd1 should negatively regulate Rac1 activity. Given that RhoGAP and Rho GTPases generally mediate signaling between cell-surface receptors and the cytoskeleton (Figure 3C), our data suggest a signaling pathway in ORN axons in which Ten-m negatively regulates Syd1, and in turn activates Rac1 GTPase for synaptic partner matching (Figure 4R).

We note that manipulating Syd1 or Rac1 levels alone did not cause significant mismatching phenotypes. These data seem to contradict a key role for Syd1 and Rac1 in regulating synaptic partner matching. A likely possibility—using Rac1 as an example—is that RNAi knockdown did not reduce the Rac1 level sufficiently to disrupt its function in promoting signals from endogenous partner recognition, but interfered with a stronger signal from overexpressed Ten-m. Two other Rac GTPases could also compensate for the Rac1 function in some developmental contexts^58,59^. Such dose-sensitive genetic interactions have been effectively used in identifying and analyzing intracellular signaling mechanisms, including those involving Rac GTPases^60–62^.

### Variations of Ten-m signaling in PN dendrites for synaptic partner matching

Given the proposed homophilic attraction between Ten-m-expressing ORN axons and PN dendrites for synaptic partner matching, we next examined Ten-m signaling mechanisms in PN dendrites. As with our approaches in ORNs, we first established a Ten-m overexpression assay in PNs and then examined genetic interactions with candidate signaling partners. We overexpressed Ten-m using *Mz19*-*GAL4*, which drives transgene expression in DA1-PNs and VA1d-PNs normally expressing low and high Ten-m, respectively, along with a marker to label their dendrites (Figure 4A). We simultaneously labeled VA1v-ORN axons, which did not intermingle with DA1- and VA1d-PN dendrites in the control (Figure 4B). However, overexpressing Ten-m in Mz19-PNs caused a partial mismatching between Mz19-PN dendrites and VA1v-ORN axons (Figure 4C), likely due to DA1-PNs with an elevated Ten-m level now matching with VA1v-ORNs, which also express high-level Ten-m^5^. This mismatching phenotype (quantified as mismatch index in Figure 4F, G) provided a quantitative assay for studying genetic interactions in PN dendrites.

Co-expression of a *Syd1*-*RNAi* transgene with Ten-m in Mz19-PNs enhanced the mismatching phenotype (Figure 4C, E, G). Conversely, co-expression of wild-type Syd1 suppressed the mismatching phenotype (Figure 4C, I, L). Expression of *Syd1*-*RNAi* or wild-type Syd1 alone did not cause mismatching (Figure 4D, G, H, L). Expression of Syd1-R979A did not affect the Ten-m overexpression phenotype (Figure 4J–L), suggesting that the suppressive effect of Syd1 depends on its RhoGAP activity. Furthermore, co-expression of *Rac1*-*RNAi* or wild-type Rac1 suppressed or enhanced the phenotype, respectively (Figure 4M–Q). Thus, a similar signaling pathway applies in PN dendrites as in ORN axons: Ten-m negatively regulates Syd1, which in turn activates Rac1 (Figure 4S, left; compared to Figure 4R).

We also uncovered differences in Ten-m signaling in PN dendrites and ORN axons. Among the Ten-m intracellular interactome (Figure 3A; Table S2) was Genghis Khan (Gek), a serine/threonine kinase previously identified as an effector of the small GTPase Cdc42^63^ (Figure S4M). Co-immunoprecipitation revealed that recombinant Ten-m and Gek interact or share a same protein complex in S2 cells, and the Ten-m-ΔECD is sufficient to mediate this interaction (Figure S4N). This prompted us to perform genetic interaction experiments in both ORN axons and PN dendrites. In ORN axons, we did not detect a significant genetic interaction between Ten-m and Gek or Gek-associated Cdc42 (Figure S4). However, in PN dendrites, Gek knockdown enhanced the Ten-m-overexpression phenotype (Figure S5A–C), whereas Gek overexpression suppressed the Ten-m-overexpression phenotype (Figure S5D, E, H). Overexpression of a kinase-dead Gek mutant (K129A)^63,64^ did not suppress the Ten-m-overexpression phenotype (Figure S5F–H), suggesting that the kinase activity is required for Gek’s function in counteracting Ten-m. Finally, Cdc42 knockdown also enhanced, whereas Cdc42 overexpression suppressed, the Ten-m-overexpression phenotype (Figure S5I–M). Thus, Gek and its upstream activator Cdc42 negatively interact with Ten-m signaling in PN dendrites but not in ORN axons (Figure 4S).

### Syd1 and Rac1 levels also modify *Ten*-*m* loss-of-function phenotypes

**Figure 5.**
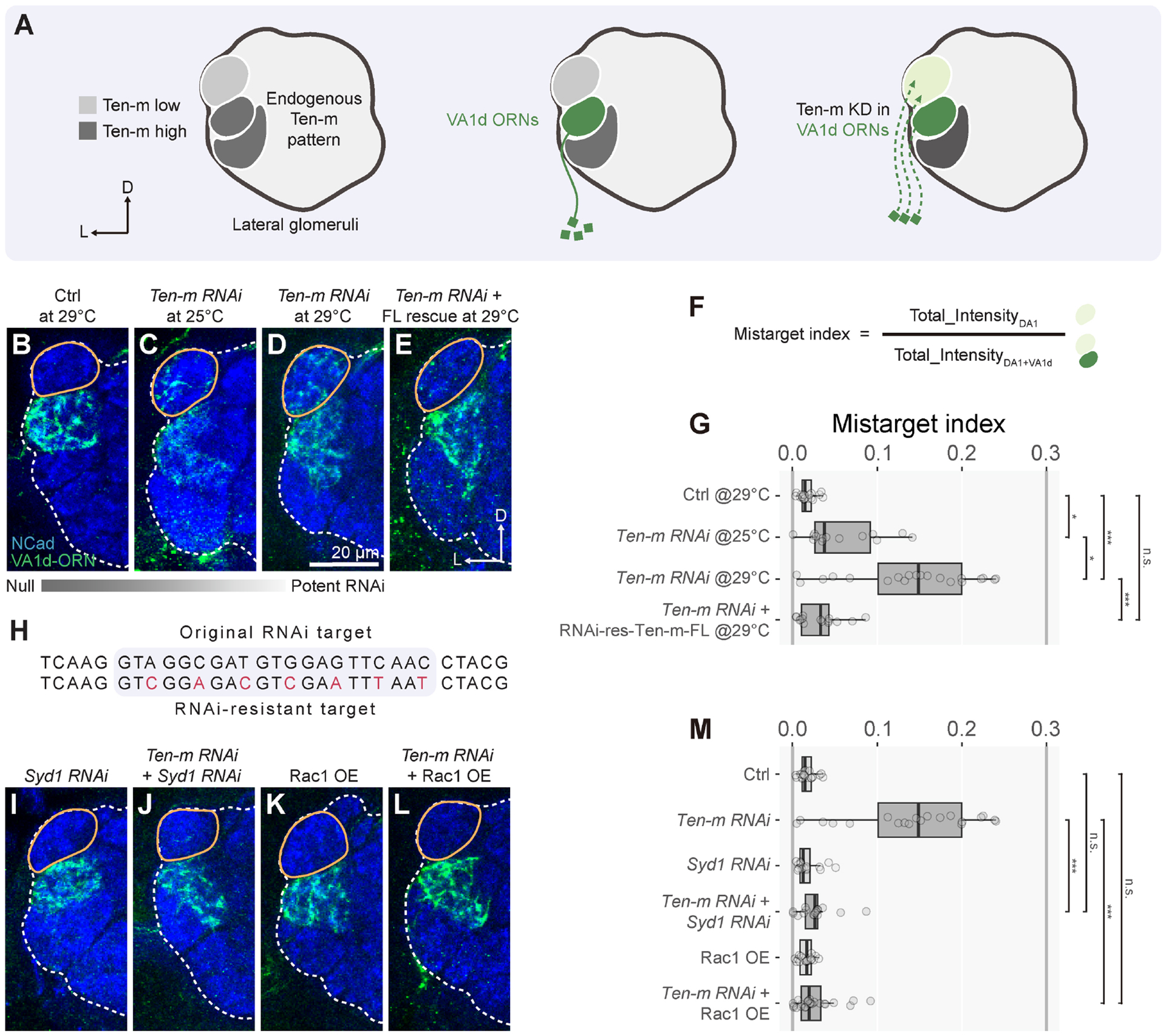
Syd1 and Rac1 modify *Ten*-*m* loss-of-function phenotypes (A) Loss-of-function assay for Ten-m signaling. Left, Ten-m expression levels. Ten-m knockdown using a split-GAL4 in VA1d-ORNs (middle) causes partial mistargeting of VA1d-ORNs to the DA1 glomerulus (right). (B–E) Representative confocal images of VA1d-ORN axons of control (B), *Ten*-*m*-*RNAi* at 25°C (C), *Ten*-*m*-*RNAi* at 29°C (D), and *Ten*-*m*-*RNAi* with RNAi-resistant full-length (FL) *Ten*-*m* rescue at 29°C (E). (F, G) Mistarget index (F) from experiments in Panels B–E (G). (H) Original and RNAi-resistant *Ten*-*m* transgene sequences at the RNAi target site. (I–M) Representative confocal images of VA1d-ORN axons of *Syd1*-*RNAi* (I), *Ten*-*m*-*RNAi* and *Syd1*-*RNAi* (J), Rac1 overexpression (K), and *Ten*-*m*-*RNAi* with Rac1 overexpression (L). Quantified in (M). Yellow circle, DA1 glomerulus. Kruskal-Wallis test with Bonferroni post-hoc correction for multiple comparisons was used in (G) and (M).

So far, all our *in vivo* genetic interaction experiments were performed in the context of Ten-m overexpression. We next examined genetic interactions in the context of *Ten*-*m* loss-of-function. We identified a split-GAL4 with an early onset expression specifically in VA1d-ORNs, which express high Ten-m^5^ (Figure 5A). Expressing *Ten*-*m*-*RNAi* in VA1d-ORNs caused a fraction of VA1d-ORN axons to innervate the neighboring DA1 glomerulus expressing low Ten-m (Figure 5B, D, F, G). Elevating the level of *Ten*-*m*-*RNAi* expression at 29°C compared to at 25°C resulted in a stronger mistargeting phenotype (Figure 5C, D, G). Furthermore, co-expression of an RNAi-resistant transgene^65^ encoding the full-length Ten-m rescued the mistargeting phenotype due to *Ten*-*m*-*RNAi* expression (Figure 5H, G). Thus, Ten-m loss in VA1d-ORNs caused a level-dependent axon mistargeting to the DA1 glomerulus.

We used this loss-of-function assay to test for genetic interactions between Ten-m and Syd1 or Rac1. While expressing *Syd1*-*RNAi* or wild-type Rac1 alone did not cause significant mismatching (Figure 5I, K, M), co-expression of *Syd1*-*RNAi* or wild-type Rac1 with *Ten*-*m*-*RNAi* suppressed mistargeting of VA1d-ORN axons to DA1 (Figure 5J, L, M). These experiments support the signaling pathway deduced from our gain-of-function genetic assay: that Ten-m negatively regulates Syd1, in turn activating the Rac1 GTPase (Figure 4R).

### Single-axon analyses support a stabilization-upon-contact model for synaptic partner matching

**Figure 6.**
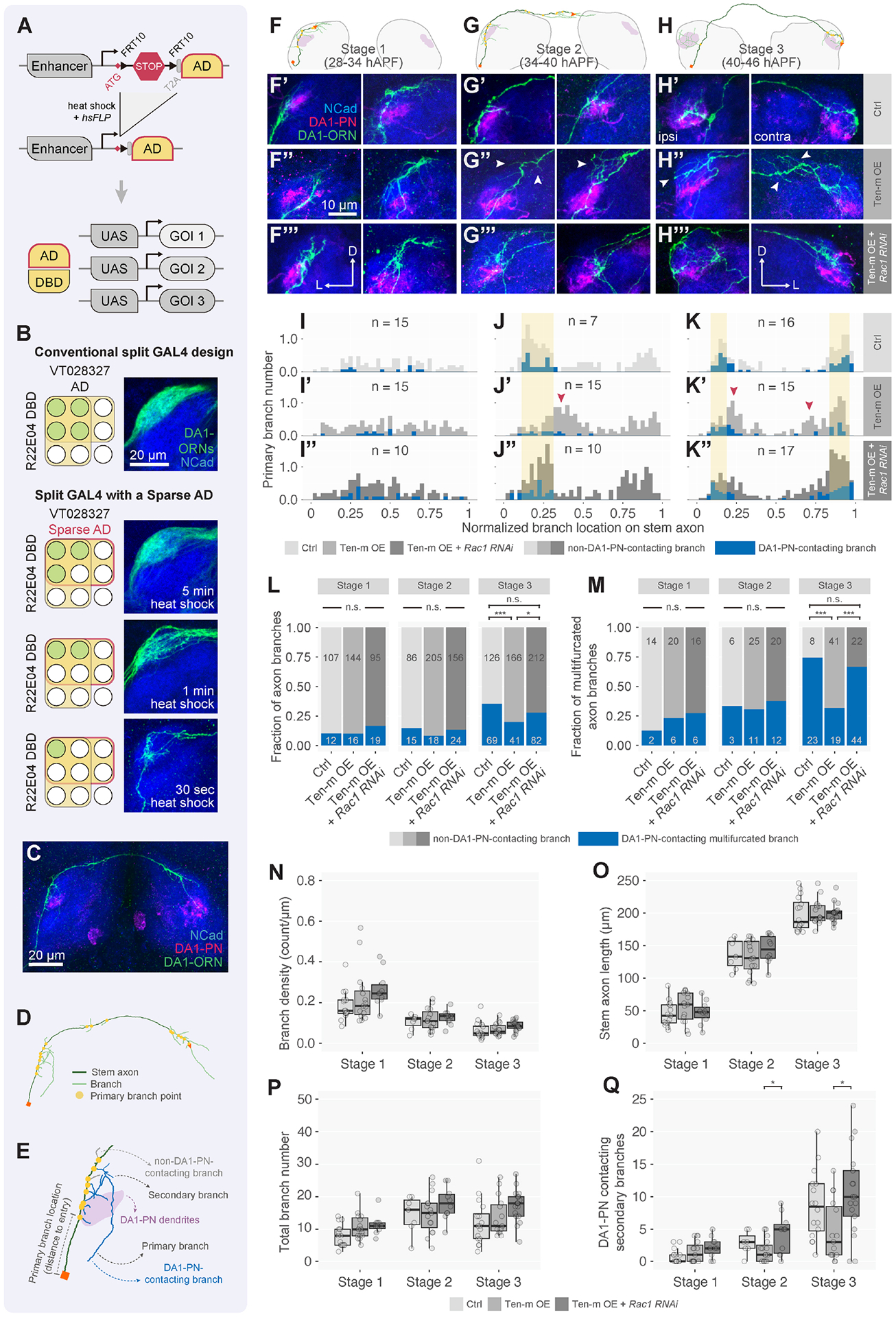
Analysis of Ten-m signaling with single-axon resolution (A) The “sparse driver” strategy. In a split-GAL4, the transcription activation domain (AD) is controlled by an enhancer separated by *FRT10*-*STOP*-*FRT10*. FLP-induced recombination between *FRT10* sites occurs at ~10% efficiency compared to wild-type *FRT* sites. STOP designates a transcription termination sequence. Heat-shock-induced FLP expression removes the STOP and enables AD expression in a fraction of cells, which together with the GAL4 DNA-binding domain (DBD) expressed from a separate transgene would reconstitute functional GAL4, driving co-expression of multiple genes of interest (GOI) in these cells. (B) Compared to conventional split-GAL4, sparse driver enables different sparsity of transgene expression tuned by heat-shock time. (C) Example of a single DA1-ORN axon innervating both ipsilateral and contralateral antennal lobes, enabled by sparse driver. (D) Z-projection of the 3D trace of the example DA1-ORN axon in (C) illustrating quantitative parameters extracted from the trace. Length of the stem axon (dark green) is measured from the antennal lobe entry point (orange square) to the end point (orange triangle). A primary branch point (yellow dot) is where a collateral branch (light green) intersects with the stem axon. (E) Zoom-in of the example DA1-ORN axon. Primary branch location is defined as the distance between the antennal lobe entry point (orange square) and the primary branch point (yellow dot). Some primary and secondary DA1-ORN branches are in contact with DA1-PN dendrites (purple shade). (F–H) Three stages of a developing DA1-ORN axon. (F) Stage 1: stem axon length <100 μm, usually before midline crossing. (G) Stage 2: stem axon length 100–170 μm; most axons have crossed the midline but have not reached the contralateral PN dendrites. (H) Stage 3: stem axon length >170 μm; most axons have reached the contralateral PN dendrites. Purple shade, DA1-PN dendrites. (F’-H”‘) Representative maximum Z-projection images of sparse DA1-ORN axons in control (F’–H’), Ten-m overexpression (F”–H”), and Ten-m overexpression with *Rac1*-*RNAi* (F’”–H’”) at each developmental stage Two examples per genotype are shown for Stages 1 and 2. For Stage 3, a single example in both ipsilateral (left) and contralateral (right) antennal lobes is shown. Arrowheads indicate dorsomedially shifted branches. (I–K”) Histograms of primary branch point distribution of DA1-ORN axons in control (I–K, top), Ten-m overexpression (I’–K’, middle), and Ten-m overexpression with *Rac1*-*RNAi* (I”–K”, bottom) at each stage. On the x-axis, 0 represents the antennal lobe entry point and 1 represents the end point of the stem axon. Right shifts of ipsilateral branches and left shifts of contralateral branches indicate dorsomedial shifting. Blue portions of the histogram indicate DA1-ORN axon branches in contact with DA1-PN dendrites. Yellow shade indicates peaks of DA1-PN contacting branches in control. Red arrowheads indicate shifted histogram peaks due to mistargeted axons. (L, M) Fractions of DA1-ORN axon branches (L) or multifurcated axon branches (M) in contact with DA1-PN dendrites. Blue and gray represent DA1-PN-contacting and non-contacting branches, respectively. A primary axon branch with at least one secondary branch is categorized as multifurcated. (N–Q) Quantification of branch densities (N), stem axon lengths (O), total branch number (P) and DA1-PN-contacting secondary branch number (Q) at each developmental stage for the listed genotypes. Chi-squared tests (L, M) and the one-way ANOVA (with Tukey’s test) (N–Q) were used for multiple comparisons. See Figure S6 for additional data.

To examine in detail how Ten-m signaling affects ORN axon behavior during each step of wiring specificity establishment, we next developed a sparse driver system to limit transgene expression to a fraction of neurons of a particular type while allowing simultaneous expression of multiple transgenes (Figure 6A, S6A, S6B). The probability of the sparse driver expression can be controlled by the FLP recombinase expression level or duration. Using a heat-shock promoter to express FLP and varying heat-shock durations, we could label a large subset, an intermediate subset, or a single DA1-ORN axon (Figure 6B, S6B–D).

Previous live-imaging experiments in the antenna-brain explant suggested that an individual ORN axon extends multiple ipsilateral branches along the main axon trunk (stem axon hereafter), with a subset subsequently stabilized^66^. In those experiments, ORN identity was determined *post hoc*, limiting assessments to few examples of any specific ORN type. Furthermore, postsynaptic targets were not labeled to assess which subset of branches were selectively stabilized. The DA1-ORN sparse driver system concomitant with labeling DA1-PN dendrites allowed us to systematically characterize the behavior of individual ORN axons during targeting selection from brains with a single DA1-ORN axon labeled (Figure 6C–E; Figure S6).

First, we sorted control samples into three developmental stages based on the stem axon length and analyzed the distribution of primary branch points along the stem axon. At Stage 1 (Figure 6F), branching points were widely distributed along the entire ipsilateral stem axon (Figure 6F’, 6I); only a small fraction of these branches contacted DA1-PN dendrites (Figure 6F’; blue in Figure 6I, L). At Stage 2 (Figure 6G), while the total primary branch density decreased compared to Stage 1 (Figure 6N), more branches contacted DA1-PN dendrites (Figure 6G’, J, L). At Stage 3 (Figure 6H), the primary branches continued to cluster near the DA1-PN dendrites (Figure 6H’, K), the primary branch density in the ipsilateral antennal lobe further decreased (Figure 6H’, left; Figure 6N), and the fraction of DA1-ORN branches contacting DA1-PN dendrites increased (Figure 6K, L). DA1-ORN axons also produced many branches in the contralateral antennal lobe, some of which contacted the contralateral DA1-PN dendrites (Figure 6H’, right; Figure 6K). Further, the number of multifurcated branches (primary branches with higher-order branches) and particularly those contacting DA1-PN dendrites increased substantially (Figure 6M, left columns).

**Figure 7.**
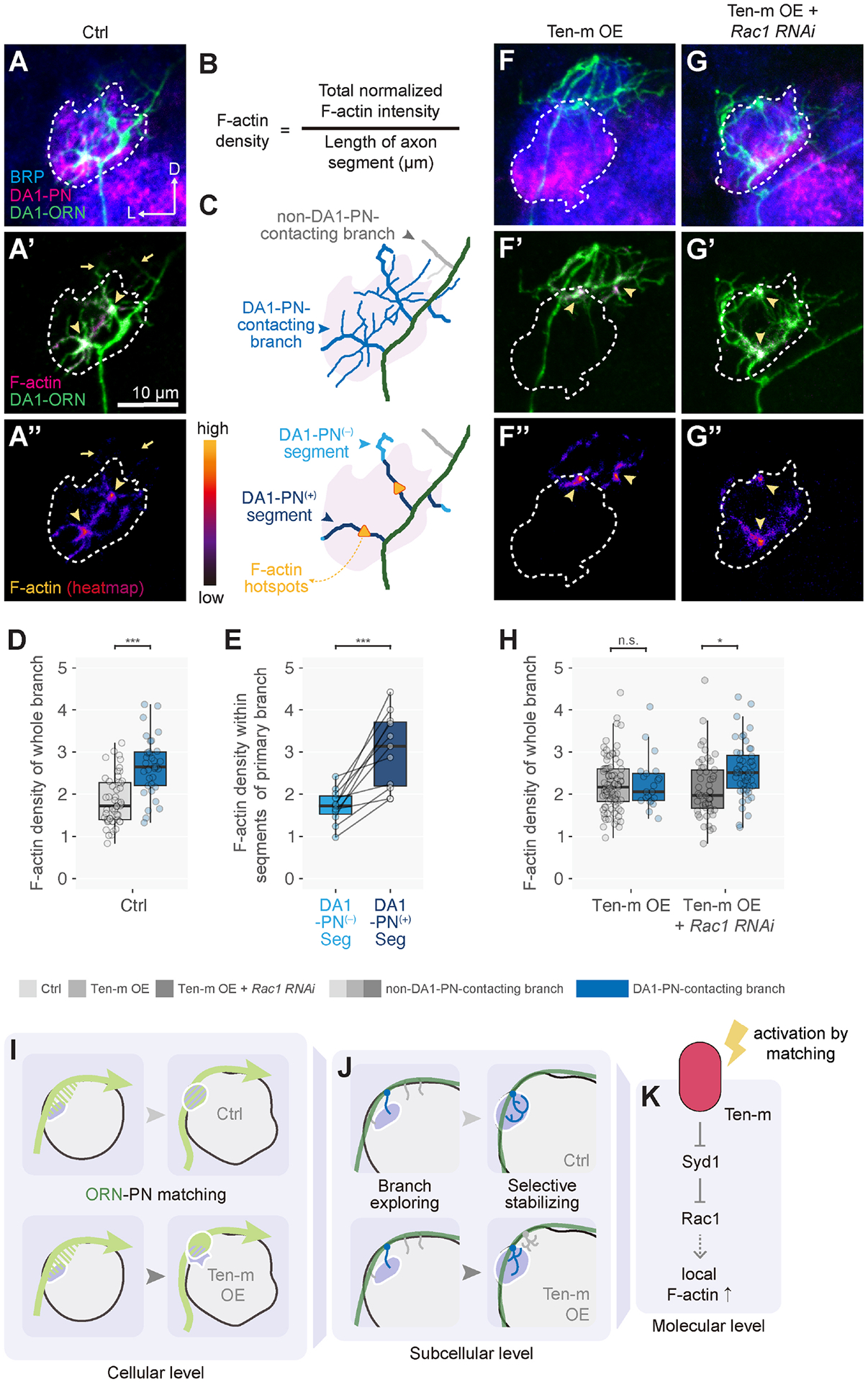
F-actin distribution analysis and summary (A–A”) Representative confocal images of DA1-PN dendrites (A, magenta), a DA1-ORN axon (A and A’, green), and F-actin distribution in the same DA1-ORN axon (A’, magenta; A”, heatmap based on Halo-Moesin staining) of control. Arrows, non-DA1-PN-contacting primary branches; arrowheads, F-actin hotspots. Dashed white traces outline DA1-PN dendrites. (B) F-actin density definition. (C) Classification of DA1-ORN axonal branches for quantification. Top: DA1-PN-contacting branches, blue; non-DA1-PN-contacting branches, gray. Primary branches have thicker width compared to high-order branches. Bottom: triangle, F-actin hotspots in primary branches; dark blue, DA1-PN-contacting segments; light blue, non-DA1-PN-contacting segments. Purple shade, DA1-PN dendrites. (D, H) F-actin density of each axon branch of control (D), Ten-m overexpression (H, left), and Ten-m overexpression with *Rac1*-*RNAi* (H, right). Each dot represents one DA1-ORN axon branch that contacts (blue) or does not contact (gray) DA1-PN dendrites. (E) F-actin densities of DA1-PN-contacting segments [DA1-PN(+)] and non-DA1-PN-contacting segments [DA1-PN(−)] in DA1-PN-contacting primary branches in control. Each dot represents one primary DA1-PN-contacting branch. (F–G”) Representative confocal images of DA1-PN dendrites, DA1-ORN axons, and F-actin distribution of Ten-m overexpression (F–F”), and Ten-m overexpression with *Rac1*-*RNAi* (G–G”). Labels same as A–A”. (I–K) Summary of the Ten-m signaling in synaptic partner matching. Ten-m level directs ORN-PN synaptic partner matching (I). Developmental single-axon analysis revealed that Ten-m specifically acts at the step of stabilizing axon branches but not general axon growth or branch exploration (J). *In situ* spatial proteomics and *in vivo* genetic perturbations delineated the signaling axis: Ten-m negatively regulates the RhoGAP Syd1, in turn activating the Rac1 GTPase to tune F-actin distribution (K). Data are from 6 axons for each genotype. Mann-Whitney *U* tests were used for comparisons (D, H). A paired *t* test was used for the within-branch comparison (E). See Figure S7 for additional data.

In summary, quantitative single-axon analyses revealed that DA1-ORN axons send many primary branches as the stem axon extends along the surface of the antennal lobe. As development proceeds, branch density decreases, branch points concentrate near DA1-PN dendrites, more branches contact PN dendrites, and more high-order branches emerge from DA1-PN dendrite contacting primary branches. These observations support a model in which stabilization of ORN axon branches by target PN dendrites is a key mechanism of target selection (Figure 7I, J).

### Ten-m signaling promotes stabilization of ORN axon branches that contact partner PN dendrites

We next probed the cellular mechanism by which perturbing Ten-m signaling affects synaptic partner matching using single-axon analysis of DA1-ORNs. We focused on two genotypes in comparison with the control: (1) Ten-m overexpression in DA1-ORNs, which caused mismatching between DA1-ORNs and DA1-PNs when assayed in bulk (Figure 1F–I), and (2) Ten-m overexpression together with RNAi against *Rac1* in DA1-ORNs, which ameliorated the mismatching phenotype caused by Ten-m overexpression (Figure 3P, S).

Compared to controls, neither experimental condition significantly affected branch density (Figure 6N), stem axon length (Figure 6O), or total branch number (Figure 6P) at all three stages. Perturbing Ten-m signaling also did not affect the distribution of axon branches along the stem axon (Figure 6F”, 6I’) or fractions of axon branches contacting DA1-PN dendrites (Figure 6F”, 6L, 6M) at early developmental stages. However, beginning at Stage 2 (Figure 6G”, 6J’) and continuing at Stage 3 (Figure 6H”, 6K’), axon branches of Ten-m-overexpressing DA1-ORNs were further from stem axon origin compared to the control, consistent with the mistargeting of axons in the bulk ORN assay (Figure 1F–H, Figure S2F–I). Rac1 knockdown in Ten-m-overexpressing DA1-ORN neurons shifted the branch distribution back to the control pattern (Figure 6G’”, 6H’”, 6J”, 6K”) and suppressed Ten-m-overexpression-induced reduction of DA1-PN-contacting ORN axon branches (Figure 6L), most strikingly for the multifurcated axons at Stage 3 (Figure 6M, 6Q).

Thus, perturbing Ten-m signaling alters neither general axon growth and branching, nor initial stages of branch exploration. Rather, Ten-m signaling promotes stabilization of ORN axon branches that contact dendrites of their partner PNs, particularly for higher-order branches. That almost all phenotypes caused by Ten-m overexpression at single-axon resolution were suppressed by reducing Rac1 level reinforces the notion that Rac1 is a key mediator of Ten-m signaling in synaptic partner selection (Figure 7J, K).

### Partner recognition promotes actin polymerization in axon branches

Given the key role of Rac1 signaling in cytoskeletal regulation^57,67–70^, we next examined microtubules and filamentous actin (F-actin) distributions using transgenic markers expressed in sparsely labeled ORNs. We found that microtubule markers—a tagged tubulin subunit^71^ or EB1 that labels growing microtubule plus ends^72^—were present along the entire length of DA1-ORN axons (Figure S7A–B’). However, Halo-Moesin, binding preferentially to F-actin^73^, preferentially localized to subcellular regions near the DA1 glomerulus (Figure S7C, C’), suggesting a role for F-actin in synaptic partner matching.

Focusing on F-actin distribution, we next examined control samples with sparsely labeled axons to resolve individual branches while co-labeling DA1-PN dendrites to determine contacts by individual ORN axon branches (Figure 7A–C). We found that DA1-ORN axon branches contacting DA1-PN dendrites had significantly higher F-actin density than those not contacting DA1-PN dendrites (Figure 7A”, D). Furthermore, within DA1-PN-contacting primary branches, segments that contacted DA1-PN dendrites had significantly higher F-actin density than segments from the same primary branches that did not contact DA1-PN dendrites (Figure 7E). These data suggest that ORN axons receive a local signal from partner PN dendrites, which promotes actin polymerization in ORN axons, potentially initiating synaptic connections.

In Ten-m-overexpressing DA1-ORNs, the F-actin density difference between branches with or without DA1-PN dendrite contact disappeared (Figure 7F”, H). This is likely because ORN branches that did not contact DA1-PN dendrites could nevertheless receive a partner matching signal from a new partner such as DL3-PNs (Figure S2), which activated Ten-m and Rac1 and thus promoted actin polymerization. In DA1-ORNs with Ten-m overexpression and Rac1 knockdown, DA1-PN-contacting branches had higher F-actin density than non-DA1-PN-contacting branches (Figure 7G”, H) again, consistent with the observation that a reduced level of Rac1 diminished the effect of Ten-m signaling and further supporting that Rac1 is the key mediator transforming Ten-m signaling into F-actin regulation (Figure 7K).

## DISCUSSION

By manipulating the levels of Ten-m, a synaptic partner matching regulator^5,6^ and following single axons of a defined neuron type across development, we showed that synaptic partner matching is primarily mediated by selective stabilization of axon branches that contact dendrites of postsynaptic partners. Combining *in situ* proximity labeling, proteomic analysis, and *in vivo* genetic interactions, we elucidated molecular pathways by which Ten-m signals to the actin cytoskeleton to mediate its function in synaptic partner matching (Figures 7I–K).

### Cellular mechanisms of synaptic partner matching

An essential step in establishing wiring specificity is to select synaptic partners among many non-partner cells. Studies of neuromuscular junctions from insects to mammals, where motor axons and their muscle targets can be readily resolved by light microscopy, suggest that axons of specific motor neuron types (or motor pools in vertebrates) navigate precisely to specific muscle targets^74–77^. However, connectivity between individual motor neurons *within the same motor pool* and specific muscle fibers *within the same muscle* in mice appears more stochastic, involving the formation of exuberant connections followed by extensive synapse elimination in an activity-dependent manner^75,78–80^. Cellular mechanisms underlying synaptic partner selection in the CNS are more difficult to discern because this involves visualizing pre- and postsynaptic partners with synaptic resolution or performing electrophysical recordings. Two best-studied systems, the climbing fiber–Purkinje cell connections and eye-specific connections between retinal ganglion cells and thalamic target neurons, both involve initially forming exuberant connections followed by activity-dependent synapse elimination^81^.

The glomerular organization of the olfactory systems provides an ideal model to investigate mechanisms of synaptic partner matching in the CNS. The convergence of axons of the same ORN type and dendrites of cognate postsynaptic partner PNs (equivalent to mitral/tufted cells in vertebrates) to discrete glomeruli allows synaptic partner matching to be examined with light microscopy, as glomerular targeting equates to synaptic partner matching. Indeed, in *Drosophila*, serial electron microscopic studies indicate that all ORN axons targeting a specific glomerulus form synaptic connections with all partner PNs^82–85^. Here, we show that after an ORN axon chooses a specific trajectory^86,87^, it produces exuberant branches followed by stabilization of those that contact dendrites of their postsynaptic partner (Figure 6). Misexpressing Ten-m, an instructive synaptic partner matching molecule and thereby partially respecifying its synaptic partners, causes stabilized axonal branches at a new target (Figure 6). Collectively, these data suggest that synaptic partner matching is largely achieved by selective axon branch stabilization resulting from molecular signaling between synaptic partners.

Our finding superficially resembles the formation of exuberant connections followed by synapse elimination in the vertebrate systems discussed above, as well as activity-dependent refinement of ORN axons and mitral cell dendrites in glomeruli of the mammalian olfactory bulb^88,89^. However, whereas the exuberant connections in the vertebrate systems last days and involve synapse formation and elimination, the exuberant ORN axon branches we observed lasted from hours to minutes (Figure 6; ref.^66^). Furthermore, the developmental timing of ORN axon target selection precedes synaptogenesis in the *Drosophila* brain^90,91^ or onset of odorant receptor expression^92^, suggesting that it is independent of synaptic or sensory activity. We propose that the exuberant ORN axon branches serve the purpose of expanding the search space for molecular interactions between ORN axons and their synaptic partners (resulting in stabilization) or non-partners (resulting in pruning). Whether a similar mechanism operates in synaptic partner matching in other circuits in the fly and vertebrate nervous systems remains an interesting question.

### Intracellular signaling mechanisms of Ten-m in synaptic partner matching

Most well-studied receptors for intercellular signaling are type-I single-pass transmembrane proteins or G-protein-coupled receptors that span the membrane 7 times^37,93–97^. Little is known about how intracellular signaling works for type-II transmembrane proteins like teneurins. Intracellular domains of teneurins do not have motifs suggestive of engaging specific signaling pathways. Thus, we took an unbiased approach of identifying potential interaction partners using proximity labeling followed by quantitative mass-spectrometry analysis, which captures both stable and transient molecular partners *in situ* in developing fly brains with a proteome-wide coverage^43^. Ten-m interactome included broad classes of proteins localized at the cell surface, synapse, cytoplasm, and endomembrane systems (Figure 2, Figure S3). Future investigation of these proteins could deepen our understanding of type-II transmembrane proteins and answer whether their inverted topology (N-terminal intracellular domain) engages distinct pathways for protein trafficking, post-translational modification, quality control, and proteolysis.

Using quantitative phenotypic assays for genetic interactions *in vivo*, we identified a key signaling pathway that links Ten-m to the actin cytoskeleton in synaptic partner matching, involving a RhoGAP and the Rac1 small GTPase (Figure 7K). This pathway is supported by genetic interaction data for both RhoGAP and Rac1, through overexpression and knockdown manipulations of RhoGAP and Rac1, in Ten-m gain-of-function and loss-of-function contexts, and in bulk and single-axon assays. Rho GTPases are key regulators of the actin cytoskeleton and have been implicated as mediators of growth cone signaling downstream of multiple classic guidance receptors, predominantly type-I transmembrane proteins^49,57,69,70,93,98,99^. That Rac1 also mediates signaling downstream of a type-II transmembrane protein, Ten-m, highlights the importance of Rho GTPases as a signaling hub.

### High-resolution methods for developmental analysis *in vivo*

In neural circuit wiring and other developmental processes, molecular signaling directs cellular behaviors. However, *in vivo* genetic analysis to interrogate functions of specific molecules and mechanistic cell biological studies are often detached from each other due to separate experimental paradigms. Our study attempts to break this barrier by developing and utilizing high-resolution methods, from spatial proteomics to single-axon analysis. Specifically, *in situ* proximity labeling with high spatiotemporal resolution and quantitative mass spectrometry enable identifying proteome-wide interacting partners of key proteins in desired biological processes, developmental stages, and subcellular locations. This can inform high-resolution phenotypic analyses and genetic interaction studies to validate the *in vivo* relevance of interacting partners.

In neural circuit development, sparse neuronal labeling and genetic manipulation allow visualizing individual neurons within dense CNS networks and the study of cell-autonomous gene function^100–105^. However, genetic manipulation methods relying on probabilistic gating of transgene expression often fail to co-express all desired genes of interest in the same sparsely labeled neurons because different effector or reporter transgenes may be stochastically expressed in independent subsets of neurons^66,106,107^. Probabilistic expression of a driver transgene, which controls the expression of multiple effector or reporter transgenes, should theoretically overcome this caveat. The MARCM system^108^ is such an example, but its reliance on the loss of a repressor after mitotic recombination limit the effectiveness of analyzing developmental events shortly after mitotic recombination because of repressor perdurance^109^.

Our sparse driver strategy (Figure 6A) achieved this by using FLPout that combines mutant *FRT* sites with reduced recombination efficiency and tunable FLP recombinase levels. Sparse expression of a transcriptional activation domain further enabled the combinatorial use with a variety of existing transgenes expressing the DNA-binding domains of transcription factors^110–112^ in specific cell types, enabling timely co-expression of multiple transgenes in cell-type-specific sparse neurons. This strategy permitted multi-parameter quantification of developing single axons while genetically manipulating Ten-m and Rac1. The combination of the above strategies can be used to dissect cellular and molecular mechanisms of other developmental processes, with the goal of integrating *in vivo* cell biology with their underlying molecular signaling cascades.

### Limitations of this study

Data from both flies and mice support that teneurins mediate homophilic attraction between axons and target neurons^5,6,13,14^. However, teneurins in principle could also mediate homophilic adhesion between axons, which may compete with axon-target interaction. How this potential competition is resolved is not known. While Ten-m’s potential role in axon-axon interaction cannot be dismissed, data obtained from our single ORN axon perturbation experiments indicate that Ten-m’s function in synaptic partner matching is mediated by ORN-PN interaction, rather than a secondary consequence of ORN axon-axon interaction. The intracellular domains of teneurins are phylogenetically diversified on amino acid sequences, so it remains to be tested whether the signaling pathways we identified apply to Ten-a in *Drosophila* and teneurins in other organisms. Since many teneurin-mediated biological processes, such as neuronal migration and synapse formation, involve extracellular interaction–modulated cellular morphogenesis, they might utilize the pathway involving Rho GTPase signaling to the actin cytoskeleton, or variations on the same theme. Finally, the biochemical mechanism by which extracellular teneurin binding inhibits RhoGAP remains a future challenge.

## STAR ★METHODS

### RESOURCE AVAILABILITY

#### Lead contact

Further information and requests for resources and reagents should be directed to the lead contact, Liqun Luo (lluo@stanford.edu).

#### Materials availability

All unique reagents generated in this study are available from the lead contact.

#### Data and code availability

- The original mass spectra and the protein sequence database used for searches have been deposited in the public proteomics repository MassIVE (https://massive.ucsd.edu) with the associated MSV identifier MSV000094010 and are accessible at ftp://massive.ucsd.edu/v07/MSV000094010/. Processed proteomic data is provided in Table S1.
- This paper does not report original code.
- Any additional information required to reanalyze the data reported in this paper is available from the lead contact upon request.

#### Experimental Model and Participant Details

##### *Drosophila* stocks and genotypes

Flies were raised on standard cornmeal medium in a 12h/12h light cycle at 25°C. To increase transgene expression, 29°C was used for some experiments as specified in the figure legend. Complete genotypes of flies in each experiment are described in Table S3. The following lines were used: *GMR22E04*-*GAL4*^*DBD*^ (the DBD of the DA1-ORN split GAL4)^113^, *VT028327*-*p65*^*AD*^ (the AD of the DA1-ORN split GAL4)^114^, *GMR31F09*-*GAL4*^*DBD*^ and *GMR78H05*-*p65*^*AD*^ (the DBD and the AD of the VA1d-ORN split GAL4)^115^, *Mz19*-*GAL4*^116^, *QUAS*-*mtdTomato*-*3xHA*^117^, *Or47b*-*rCD2*^118^, *trans*-Tango^42^, *UAS*-*dcr2*^119^, *UAS*-*mCD8*-*GFP*^108^, *hsFLP* (*heat shock protein* promoter-driven *FLP*)^120^, *NP*-*6658*-*GAL4* (*Ten*-*m*-*GAL4*)^5^*, P{GS}9267 (UAS*-gated *Ten*-*m* overexpression), and *QUAS*-*Ten*-*m*^5^, and *UAS*-*myr*-*mGreenLantern*^39^. *UAS*-*Syd1*-*WT*-*3xFLAG* and *UAS*-*Syd1*-*R979A*-*3xFLAG*^56^ were kindly provided by the Herman lab (University of Oregon), *UAS*-*Gek and UAS*-*Gek*-*K129A*^64^ used for early experiments were kindly provided by the Clandinin lab (Stanford University). The DA1-ORN lines, one with Ten-m overexpression and one without (Figure 1, S1, 3, and S4), were generated in this study. The Mz19-PN line with Ten-m overexpression (Figure 4) was generated based on the previously built Mz19-PN genetic screen line^5^. The VA1d-ORN line (Figure 5) is an unpublished reagent generously provided by Cheng Lyu. *Mz19*-*QF2*^*G4HACK*^*, UAS*-*Halo*-*alphaTub84B, UAS*-*Halo*-*EB1*, *UAS*-*Halo*-*Moesin*, *UAS*-*V5*-*Ten*-*m*, *UAS*-*V5*-*Ten*-*m*-*ΔECD*, *UAS*-*V5*-*Ten*-*m*-*ΔICD*, *APEX2*-*V5*-*Ten*-*m, UAS*-*CD4*-*APEX2*, *UAS*-*Gek*-*FLAG, UAS*-*Gek*-*K129A*-*FLAG*, *UAS*-*V5*-*Ten*-*m (RNAi*-*resistant)*, and *VT028327*-*FRT10*-*STOP*-*FRT10*-*p65AD* were generated in this study. The other RNAi or overexpression lines were generated previously^119,121,122^ and acquired from the Bloomington *Drosophila* Stock Center and the Vienna *Drosophila* Resource Center (stock numbers listed in Table S3). Similar knockdown effects were observed by multiple RNAi transgenes targeting non-overlapping regions of each gene.

### METHOD DETAILS

#### Generation of *APEX2*-*V5*-*Ten*-*m* flies

The *APEX2*-*V5*-*Ten*-*m* fly line was generated by CRISPR-mediated knock-in to the *Ten*-*m* genomic locus. Briefly, to build the homology-directed repair (HDR) vector, a ~1500bp genomic sequence flanking the *Ten*-*m* start codon (~750bp each side) was amplified using the Q5 hot-start high-fidelity DNA polymerase (New England Biolabs) and inserted into the *pCR*-*Blunt*-*TOPO* vector (Thermo Fisher). The codon-optimized APEX2-V5 sequence was synthesized as a gBlock (Integrated DNA Technologies) and inserted into the *TOPO genomic sequence plasmid* using the NEBuilder HiFi DNA assembly master mix (New England Biolabs). CRISPR guide RNA (gRNA) targeting a locus near the start codon was designed using the flyCRISPR Target Finder web tool^123–125^ and cloned into the *pU6*-*BbsI*-*chiRNA* vector^126^ (Addgene #45946) by NEBuilder HiFi DNA assembly master mix. Silent mutations were introduced at the PAM site of the HDR vector by using the Q5 site-directed mutagenesis kit (New England Biolabs). The *APEX2*-*V5*-*Ten*-*m* HDR and the *Ten*-*m* gRNA vectors were co-injected into *vas*-*Cas9*^127^ fly embryos by BestGene. G0 flies were crossed to a third chromosome balancer line and all progenies were individually balanced and genotyped until APEX2-insertion-positive candidates were identified. APEX2-insertion-positive candidates were sequenced and then kept.

*APEX2*-*V5*-*Ten*-*m* allele did not appear to interfere with Ten-m function wiring of the olfactory circuit in the antennal lobe, as homozygous flies (1) were viable as opposed to embryonic lethal for a *Ten*-*m* loss-of-function allele^11^, (2) recapitulated normal Ten-m expression patterns (Figure 2E), (3) did not affect the antennal lobe morphology and glomerular position (Figure 2C–E), and (4) showed normal wiring patterns in an assay sensitive to detect wiring defects of ORN and PN types in this study. However, we cannot rule out the possibility that we might have missed an essential partner for Ten-m’s function that we did not examine.

#### Generation of UAS constructs and transgenic flies

To generate the *UAS*-*CD4*-*APEX2*-*V5* construct, the signal peptide from the Drosophila *akh* gene, the CD4 coding sequence from *UAS*-*CD4*-*GFP*^128^, and the codon-optimized APEX2-V5 sequence (see above) were amplified using the Q5 hot-start high-fidelity DNA polymerase (New England Biolabs) and inserted into the *pJFRC81*-*10xUAS*-*IVS*-*Syn21*-*GFP*-*p10*^129^ vector (Addgene #36432) to replace the GFP sequence using NEBuilder HiFi DNA assembly master mix (New England Biolabs).

To generate the *UAS*-*Gek*-*FLAG* and *UAS*-*Syd1*-*FLAG* constructs, we extracted the total RNA of *w1118* pupal fly heads using an RNA mini-prep kit (Zymo Research), synthesized the complementary DNA using the SuperScript III First-Strand Synthesis SuperMix (Thermo Fisher), and amplified the *Gek* or *Syd1* coding sequences using the Q5 hot-start high-fidelity DNA polymerase (New England Biolabs). The verified coding sequences were then assembled into a modified *pUAST*-*attB* vector, in which a FLAG tag was added at the 3’ end.

To generate the *UAS*-*Gek*-*K129A*-*FLAG* construct, the *K129A* mutation was introduced using the Q5 site-directed mutagenesis kit (New England Biolabs).

To generate the *UAS*-*V5*-*Ten*-*m* construct, a V5 tag was inserted after the start codon of Ten-m cDNA (isoform B) in the plasmid *pUAST*-*attB*-*Ten*-*m*^5^ using the Q5 site-directed mutagenesis kit (New England Biolabs). To generate the *UAS*-*V5*-*Ten*-*m*-*ΔICD* and *UAS*-*V5*-*Ten*-*m*-*ΔECD* constructs, N2-A225 and I256-A2731 were deleted using the NEBuilder HiFi DNA assembly master mix (New England Biolabs), respectively.

To generate the RNAi^VDRC330540^-resistant *UAS*-*V5*-*Ten*-*m* construct, mutations (Figure 5I) were introduced using the Q5 site-directed mutagenesis kit (New England Biolabs).

To generate the *UAS*-*V5*-*Ten*-*m*-*FLAG*, *UAS*-*V5*-*Ten*-*m*-*ΔICD*-*FLAG*, and *UAS*-*V5*-*Ten*-*m*-*ΔECD*-*FLAG* constructs, a FLAG tag was inserted before the stop codon of the corresponding V5-tagged constructs described above.

To generate the *VT02832*-*p65AD* construct, VT027328 primers^114^ were used to amplify the sequence from the genomic DNA of *VT027328*-*p65AD* fly line (BDRC #73064) The verified sequence was then assembled into the pENTR/D-TOPO vector (Thermo Fisher) and integrated into the pBPp65ADZpUw vector using the Gateway LR Clonase II Enzyme mix (Thermo Fisher).

To generate the *VT028327*-*FRT10*-*STOP*-*FRT10*-*p65AD* construct, the *FRT10*-*STOP*-*FRT10* sequence^66^ and the T2A element were inserted after the p65AD start codon of the *VT02832*-*p65AD* construct. Each plasmid was verified by full-length DNA sequencing.

To generate *UAS*-*Halo*-*Moesin*, *UAS*-*Halo*-*EB1*, and *UAS*-*Halo*-*alphaTub84B*, the moesin actin binding domain, EB1, and alphaTub84B coding sequences were PCR amplified from the genomic DNA of transgenic flies *UAS*-*GMA* (BDRC #31775), *UAS*-*EB1*-*GFP* (BDRC #35512), and *UAS*-*GFP*-*alphaTub84B* (BDRC #7373), respectively, and subcloned into *UAS*-*Halo*-*CAAX* (Addgene #87645) using XhoI and XbaI. The *pUAST*-*attB* constructs were inserted into the *attP24* (for *Gek* constructs and *UAS*-*CD4*-*APEX2*), *VK00027* (for *VT028327*-*FRT10*-*STOP*-*FRT10*-*p65AD*), *VK00019* (for cytoskeleton marker constructs), or *attP86Fb* (for *Ten*-*m* constructs) landing sites.

Transgenic flies were generated in house by standard methods involving microinjection of DNA into early *Drosophila* embryos prior to cellularization. G0 flies were crossed to a *white*^−^ balancer, and all *white*^*+*^ progenies were individually balanced and verified.

Abbreviations of Ten-m domains (Figure 1J): EGF, epidermal growth factor repeat; CRD, cysteine-rich domain; TTR, transthyretin-related domain; Ig-like, immunoglobulin-like domain; NHL, a domain named after homology between NCL-1, HT2A, and Lin-41, also called β-propeller domain; YD-shell, enriched for tyrosine and aspartate, also called β-barrel domain; Tox-GHH, toxin-like domain.

#### Isoforms of Ten-m in overexpression experiments

According to the FlyBase, *Ten*-*m* has 3 isoforms including isoform B (FlyBase ID: FBpp0078161, RefSeq ID: NP_524215), D (FlyBase ID: FBpp0297244, RefSeq ID: NP_001097661), and E (FlyBase ID: FBpp0303192, RefSeq ID: NP_001262211). The isoform used in our UAS-cDNA-based overexpression experiments (Figure 1, 3, 5, 6, and 7) was isoform B, as its homophilic attraction function was genetically and biochemically validated in our previous study^5^. The isoform used in our EP-line-based overexpression experiments in PNs was not determined, as this strategy has the UAS element inserted at the 5’ upstream of the *Ten*-*m* genomic locus and it could drive the chosen cell type’s preferred isoform to express in the overexpression experiments (Figure 4 and ref.^5^).

#### APEX2-mediated proximity biotinylation in fly brains

The proximity labeling reaction was performed following the previously published method^46^. Briefly, we dissected APEX2-Ten-m group, spatial reference group, and negative control group in pre-chilled Schneider’s medium (Thermo Fisher) and transferred them into 500 μL of the Schneider’s medium in 1.5 mL protein low-binding tubes (Eppendorf) on ice. Brains were washed with the Schneider’s medium to remove fat bodies and debris and were incubated in 100 μM of biotin-phenol (BP; APExBIO) in the Schneider’s medium on ice for one hour, with occasional pipetting for mixing. Brains were then labeled with 1 mM (0.003%) H_2_O_2_ (Thermo Fisher) for 1 minute, and immediately quenched by five thorough washes using the quenching buffer that contains 10 mM sodium ascorbate (Spectrum Chemicals), 5 mM Trolox (Sigma-Aldrich), and 10 mM sodium azide (Sigma-Aldrich) in phosphate buffered saline (PBS; Thermo Fisher). After the washes, the quenching solution was removed, and brains were either fixed for immunostaining (see below for details) or were frozen in liquid nitrogen and stored at −80°C for proteomic analysis. For proteomic sample collection, 900 dissected and biotinylated brains were collected for each experimental group (5400 brains in total).

#### Enrichment of biotinylated proteins

Brains were processed in the original collection tube, to avoid loss during transferring. We added 40 μL of high-SDS RIPA (50mM Tris-HCl [pH 8.0], 150 mM NaCl, 1% sodium dodecyl sulfate [SDS], 0.5% sodium deoxycholate, 1% Triton X-100, 1x protease inhibitor cocktail [Sigma-Aldrich], and 1 mM phenylmethylsulfonyl fluoride [PMSF; Sigma-Aldrich]) to each tube of frozen brains, and grinded the samples on ice using disposable pestles with an electric pellet pestle driver. Tubes containing brain lysates of the same group were spun down, merged, and rinsed with an additional 100 μL of high-SDS RIPA to collect remaining proteins. Samples were then vortexed briefly, sonicated twice for ten seconds each, and incubated at 95°C for five minutes to denature proteins. 1.2 mL of SDS-free RIPA buffer (50 mM Tris-HCl [pH 8.0], 150 mM NaCl, 0.5% sodium deoxycholate, 1% Triton X-100, 1x protease inhibitor cocktail, and 1 mM PMSF) were added to each sample, and the mixture was rotated for two hours at 4°C. Lysates were then diluted with 200 μL of normal RIPA buffer (50 mM Tris-HCl [pH 8.0], 150 mM NaCl, 0.2% SDS, 0.5% sodium deoxycholate, 1% Triton X-100, 1x protease inhibitor cocktail, and 1 mM PMSF), transferred to 3.5 mL ultracentrifuge tubes (Beckman Coulter), and centrifuged at 100,000 g for 30 minutes at 4°C. 1.5 mL of the supernatant was carefully collected for each sample. 400 μL of streptavidin magnetic beads (Pierce) washed twice using 1 ml RIPA buffer were added to each of the post-ultracentrifugation brain lysates. The lysate and the streptavidin bead mixture were left to rotate at 4°C overnight. On the following day, beads were washed twice with 1 mL RIPA buffer, once with 1 mL of 1 M KCl, once with 1 mL of 0.1 M Na_2_CO_3_, once with 1 mL of 2 M urea in 10 mM Tris-HCl (pH 8.0), and again twice with 1 mL RIPA buffer. The beads were resuspended in 1 mL fresh RIPA buffer. 35 μL of the bead suspension was taken out for western blot, and the rest proceeded to on-bead digestion.

#### Western blotting of biotinylated proteins

Biotinylated proteins were eluted from streptavidin beads by the addition of 20 μL of elution buffer (2X Laemmli sample buffer [Bio-Rad], 20 mM dithiothreitol [Sigma-Aldrich], and 2 mM biotin [Sigma-Aldrich]) followed by a 10 min incubation at 95°C. Proteins were resolved by 4%–12% Bis-Tris PAGE gels (Thermo Fisher) and transferred to nitrocellulose membranes (Thermo Fisher). After blocking with 3% bovine serum albumin (BSA) in Tris-buffered saline with 0.1% Tween 20 (TBST; Thermo Fisher) for 1 hour, membrane was incubated with 0.3 mg/mL HRP-conjugated streptavidin for one hour. The Clarity Western ECL blotting substrate (Bio-Rad) and ChemiDoc imaging system (Bio-Rad) were used to develop and detect chemiluminescence.

#### On-bead trypsin digestion of biotinylated proteins

The streptavidin-enriched sample (400 μL of streptavidin beads per condition) was processed for on-bead digestion and TMT labeling and used for mass spectrometry analysis as previously described^46^. Proteins bound to streptavidin beads were washed twice with 200 μL of 50 mM Tris-HCl buffer (pH 7.5), followed by two washes with 2 M urea/50 mM Tris (pH 7.5) buffer in fresh tubes. The final volume of 2 M urea/50 mM Tris (pH 7.5) buffer was removed, and beads were incubated with 80 μL of 2 M urea/50 mM Tris buffer containing 1 mM dithiothreitol (DTT) and 0.4 μg trypsin. Beads were incubated in the urea/trypsin buffer for 1 hour at 25°C while shaking at 1000 revolutions per minute (rpm). After 1 hour, the supernatant was removed and transferred to a fresh tube. The streptavidin beads were washed twice with 60 μL of 2 M urea/50 mM Tris (pH 7.5) buffer and the washes were combined with the on-bead digest supernatant. The eluate was reduced with 4 mM DTT for 30 min at 25°C with shaking at 1000 rpm. The samples were alkylated with 10 mM iodoacetamide and incubated for 45 min in the dark at 25°C while shaking at 1000 rpm. An additional 0.5 μg of trypsin was added to the sample and the digestion was completed overnight at 25°C with shaking at 700 rpm. After overnight digestion, the sample was acidified (pH < 3) by adding formic acid (FA) such that the sample contained 1% FA. Samples were desalted on C18 StageTips (3M). Briefly, C18 StageTips were conditioned with 100 μL of 100% MeOH, 100 μL of 50% MeCN/0.1% FA, and 2x with 100 μL of 0.1% FA. Acidified peptides were loaded onto the conditioned StageTips, which were subsequently washed 2 times with 100 μL of 0.1% FA. Peptides were eluted from StageTips with 50 μL of 50% MeCN/0.1% FA and dried to completion.

#### TMT labeling and StageTip peptide fractionation

Desalted peptides were labeled with TMT6 reagents (Thermo Fisher Scientific) as directed by the manufacturer. Peptides were reconstituted in 100 μL of 50 mM HEPES. Each 0.8 mg vial of TMT reagent was reconstituted in 41 μL of anhydrous acetonitrile and added to the corresponding peptide sample for 1 hour at room temperature shaking at 1000 rpm. Labeling of samples with TMT reagents was completed with the design described in Figure 2F. TMT labeling reactions were quenched with 8 μL of 5% hydroxylamine at room temperature for 15 min with shaking. The entirety of each sample was pooled, evaporated to dryness in a vacuum concentrator, and desalted on C18 StageTips as described above. One SCX StageTip was prepared per sample using 3 plugs of SCX material (3M) topped with 2 plugs of C18 material. StageTips were sequentially conditioned with 100 μL of MeOH, 100 μL of 80% MeCN/0.5% acetic acid, 100 μL of 0.5% acetic acid, 100 μL of 0.5% acetic acid/500mM NH_4_AcO/20% MeCN, followed by another 100 μL of 0.5% acetic acid. Dried sample was re-suspended in 250 μL of 0.5% acetic acid, loaded onto the StageTips, and washed twice with 100 μL of 0.5% acetic acid. Sample was transeluted from C18 material onto the SCX with 100 μL of 80% MeCN/0.5% acetic acid, and consecutively eluted using 3 buffers with increasing pH—pH 5.15 (50mM NH_4_AcO/20% MeCN), pH 8.25 (50mM NH_4_HCO_3_/20% MeCN), and finally pH 10.3 (0.1% NH_4_OH, 20% MeCN). Three eluted fractions were re-suspended in 200 μL of 0.5% acetic acid to reduce the MeCN concentration and subsequently desalted on C18 StageTips as described above. Desalted peptides were dried to completion.

#### Liquid chromatography and mass spectrometry

Desalted TMT-labeled peptides were resuspended in 9 μL of 3% MeCN, 0.1% FA and analyzed by online nanoflow liquid chromatography tandem mass spectrometry (LC-MS/MS) using a Q Exactive Plus (for fractionated samples) (Thermo Fisher Scientific) coupled on-line to a Proxeon Easy-nLC 1200 (Thermo Fisher Scientific). 4 μL of each sample were loaded at 500 nL/min onto a microcapillary column (360 μm outer diameter × 75 μm inner diameter) containing an integrated electrospray emitter tip (10 mm), packed to approximately 28 cm with ReproSil-Pur C18-AQ 1.9 mm beads (Dr. Maisch GmbH) and heated to 50°C. The HPLC solvent A was 3% MeCN, 0.1% FA, and the solvent B was 90% MeCN, 0.1% FA. Peptides were eluted into the mass spectrometer at a flow rate of 200 nL/min. The SCX fractions were run with 110-minute method, which used the following gradient profile: (min:%B) 0:2; 1:6, 85:30; 94:60; 95:90, 100:90,101:50,110:50 (the last two steps at 500 nL/min flow rate). The Q Exactive Plus was operated in the data-dependent mode acquiring HCD MS/MS scans (r = 17,500) after each MS1 scan (r = 70,000) on the top 12 most abundant ions using an MS1 target of 3E6 and an MS2 target of 5E4. The maximum ion time utilized for MS/MS scans was 105 ms; the HCD normalized collision energy was set to 31; the dynamic exclusion time was set to 30 s, and the peptide match was set to “preferred” and isotope exclusion functions were enabled. Charge exclusion was enabled for charge states that were unassigned, 1, 7, 8, >8.

#### Mass spectrometry data processing

Collected data were analyzed using the Spectrum Mill software package (proteomics.broadinstitute.org). Nearby MS scans with a similar precursor m/z were merged if they were within ±60 s retention time and ±1.4 m/z tolerance. MS/MS spectra were excluded from searching if they failed the quality filter by not having a sequence tag length 0 or did not have a precursor MH+ in the range of 750–4000. All extracted spectra were searched against a UniProt database containing *Drosophila melanogaster* reference proteome sequences. Search parameters included: ESI QEXACTIVE-HCD-v2 scoring parent and fragment mass tolerance of 20 ppm, 40% minimum matched peak intensity, trypsin allow P enzyme specificity with up to two missed cleavages, and calculate reversed database scores enabled. Fixed modifications were carbamidomethylation at cysteine. TMT labeling was required at lysine, but peptide N termini were allowed to be either labeled or unlabeled. Allowed variable modifications were protein N-terminal acetylation and oxidized methionine. Individual spectra were automatically assigned a confidence score using the Spectrum Mill auto-validation module. Score at the peptide mode was based on a target-decoy false discovery rate (FDR) of 1%. Protein polishing auto-validation was then applied using an auto thresholding strategy. Relative abundances of proteins were determined using TMT reporter ion intensity ratios from each MS/MS spectrum and the median ratio was calculated from all MS/MS spectra contributing to a protein subgroup. Proteins identified by 2 or more distinct peptides and ratio counts were considered for the dataset.

#### Linear model for the mass spectrometry data

Starting with the processed mass spectrometry data, we developed a linear model to identify prospective interacting partners of Ten-m. Using the log_2_ transformed TMT ratios, the linear model is as follows:

$${\text{log}}_{2}\left(\text{TMT}\;\text{ratio}\right)={\text{b}}_{0}+{\text{b}}_{1}\;{\text{TRT}+\text{b}}_{2}\;\text{SR}$$

where TRT and SR are indicator variables representing APEX2-Ten-m enrichment and spatial reference, respectively. The negative control NC constitutes the baseline for the model. The [Ten-m/SR fold change] taking negative controls into account is represented by the (b_1_ – b_2_) contrast while the [Ten-m/NC fold change] is captured by the b_1_ coefficient. The model is fitted using an empirical Bayes approach and the relevant contrasts/coefficients are subject to a moderated t-test to determine nominal *p*-values for each protein in the TMT dataset. These nominal *p*-values are then corrected for multiple testing using the Benjamini-Hochberg FDR (BH-FDR) method^130^. The linear model along with the associated moderated t-test and BH-FDR correction were implemented using the limma library^131^ in R.

#### Proteomic data analysis

To identify prospective interacting partners of Ten-m, we implemented three filtering steps: (1) From the total of 3454 proteins detected across 6 samples, we focused on those with at least two unique peptides, narrowing the list down to 2854 proteins. (2) We then filtered out potential contaminants, including endogenously biotinylated and endogenous peroxidase-labeled proteins, by using the [APEX2-Ten-m/NC] fold change of the Ten-m protein itself as a threshold, resulting in 781 proteins. (3) Finally, to exclude generic proteins located near the cell membrane, we employed a [APEX2-Ten-m/SR] fold change–based ratiometric approach, isolating 294 proteins specifically enriched by APEX2-Ten-m. Functional enrichment analyses, including Gene Ontology, protein domain (SMART), reactome pathway, and local network cluster, were performed on these gene sets using the STRING database.

#### Immunocytochemistry

Fly brains were dissected and immunostained according to the previously published protocol^132^. Briefly, brains were dissected in pre-cooled PBS (phosphate buffered saline; Thermo Fisher) and then fixed in 4% paraformaldehyde (Electron Microscopy Sciences) in PBS with 0.015% Triton X-100 (Sigma-Aldrich) for 20 minutes (15 minutes for sparse axon experiments to prevent over-fixation background) on a nutator at room temperature. Fixed brains were washed with PBST (0.3% Triton X-100 in PBS) four times, each time nutating for 15 minutes. The brains were then blocked in 5% normal donkey serum (Jackson ImmunoResearch) in PBST for 1 hour at room temperature or overnight at 4°C on a nutator. Primary antibodies were diluted in the blocking solution and incubated with brains for 36–48 hours on a 4°C nutator. After washed with PBST four times, each time nutating for 20 minutes, brains were incubated with secondary antibodies diluted in the blocking solution and nutated in the dark for 24–48 hours at 4°C. Brains were then washed again with PBST four times, each time nutating for 20 minutes. Immunostained brains were mounted with the SlowFade antifade reagent (Thermo Fisher) and stored at 4°C before imaging.

Primary antibodies used in immunostaining include: rat anti-NCad (1:40; DN-Ex#8, Developmental Studies Hybridoma Bank), mouse anti-BRP (1:80; nc82, Developmental Studies Hybridoma Bank), chicken anti-GFP (1:1000; GFP-1020, Aves Labs), rabbit anti-DsRed (1:500; 632496, Clontech), rabbit anti-HA (1:100, 3724S, Cell Signaling), mouse anti-rat CD2 (1:200; OX-34, Bio-Rad), mouse anti-V5 (1:200; R960–25, Thermo Fisher), and rat anti-V5 (1:200; ab206571, Abcam). Donkey secondary antibodies conjugated to Alexa Fluor 405/488/568/647 (Jackson ImmunoResearch or Thermo Fisher) were used at 1:250. Neutravidin pre-conjugated with Alexa Fluor 647 (1:1000; synthesized in the Ting lab) was used to detect biotin.

#### HaloTag labeling

Fly brains were labelled according to the previously published protocol^133^. Janelia Fluor (JF) HaloTag dyes (stocks at 1 mM) were gifts from the Lavis lab^134,135^. Briefly, fly brains were dissected in pre-cooled PBS and then fixed in 4% paraformaldehyde in PBS for 10 minutes on a nutator at room temperature. Fixed brains were washed with PBST for 5 min, repeated 3 times, followed by incubation with JF646-HaloTag ligand (1:2000 diluted in PBS) for 5 h or overnight at room temperature in the dark. Brains were then washed with PBST for 5 min, repeated 3 times, followed by immunostaining protocol if necessary.

#### Transfection and immunostaining of *Drosophila* S2 cells

S2 cells (Thermo Fisher) were cultured in the Schneider’s medium (Thermo Fisher) following the manufacturer’s protocol. S2 cells were transfected with *Actin*-*GAL4*, along with *UAS*-*V5*-*Ten*-*m*-*FLAG*, *UAS*-*V5*-*Ten*-*m*-*ΔICD*-*FLAG*, or *UAS*-*V5*-*Ten*-*m*-*ΔECD*-*FLAG* constructs using the FuGENE HD transfection Reagent (Promega). After 48 hours, transfected cells were transferred to coverslips pre-coated with Concanavalin A (Sigma-Aldrich). For the plasma membrane non-permeabilized condition, S2 cells were incubated with rat anti-V5 antibody (1:200; Abcam) and mouse anti-FLAG M2 antibody (1:200; Sigma-Aldrich) diluted in the Schneider’s medium (Thermo Fisher) at room temperature for 1 hour. S2 cells were rinsed with PBS, fixed with 4% PFA in PBST, washed with PBST, blocked with 5% normal donkey serum (Jackson ImmunoResearch) in PBST, incubated with secondary antibodies in the dark, washed with PBST, mounted, and imaged. For the plasma membrane permeabilized condition, S2 cells were incubated in the Schneider’s medium at room temperature for 1 hour, rinsed with PBS, fixed with 4% PFA in PBST, washed with PBST, blocked with 5% normal donkey serum in PBST, incubated with primary antibodies, washed with PBST, incubated with secondary antibodies in the dark, washed with PBST, mounted, and imaged.

#### Image acquisition and processing

Images were obtained using laser scanning confocal microscopy (Zeiss LSM 780 or LSM 900). Brightness and contrast adjustments as well as image cropping were done using ImageJ.

#### Co-immunoprecipitation assay

S2 cells (Thermo Fisher) were cultured in the Schneider’s medium (Thermo Fisher) following the manufacturer’s protocol. S2 cells were transfected with *UAS*-*Syd1*-*FLAG* or *UAS*-*Gek*-*FLAG*, along with a *Ten*-*m* expression construct and *Actin*-*GAL4* using the FuGENE HD transfection reagent (Promega). After 72 hours, the transfected cells were harvested, rinsed with PBS, lysed in the lysis buffer (50 mM Tris-HCl [pH 7.5], 150 mM NaCl, 0.2% TritonX-100, 10% glycerol) supplemented with protease inhibitor cocktail (Promega). The cell lysates were rotated at 4°C for 2 hours and then centrifuged at 15,000 g for 20 minutes at 4°C. The supernatants were collected and incubated with Dynabeads Protein G beads (Thermo Fisher) pre-coated with the mouse anti-V5 antibody (1:100; R960–25, Thermo Fisher) and then left to rotate at 4°C overnight. On the following day, the samples were washed extensively in wash buffer (50 mM Tris-HCl [pH 7.5], 150 mM NaCl, 0.5% TritonX-100) for three times, 10 minutes each. The proteins were eluted from beads by adding the loading buffer (4X Laemmli sample buffer [Bio-Rad] with 20 mM dithiothreitol) followed by a 10 min incubation at 95°C. The samples were loaded in 3%–8% Tris-Acetate PAGE gels (Thermo Fisher) for protein electrophoresis and transferred to PVDF membranes (Thermo Fisher) at 15V overnight. The membranes were blocked with the SuperBlock blocking buffer (Thermo Fisher), incubated with mouse anti-FLAG M2 antibody (1:3000; Sigma-Aldrich), washed with TBST (Thermo Fisher), incubated with light chain specific HRP-conjugated secondary antibodies (1:5000; Jackson ImmunoResearch), washed with TBST, and developed with Clarity Western ECL blotting substrate (Bio-Rad).

#### Sparse axon labeling and genetic manipulation

Each fly contains the DA1-ORN sparse driver and its reporter (*UAS*-*myr*-*mGreenLantern, UAS*-*mCD8*-*GFP*, *VT028327*-*FRT10*-*STOP*-*FRT10*-*p65AD*, *GMR22E04*-*GAL4*^*DBD*^), *hsFLP*, the DA1-PN driver and its reporter (*Mz19*-*QF2*^*G4HACK*^, *QUAS*-*mtdTomato*-*3xHA*), and other desired UAS constructs for genetic manipulation (*UAS*-*V5*-*Ten*-*m or UAS*-*dcr2*, *UAS*-*Rac1*-*RNAi*^*BDRC28985*^). For sparse axon experiments imaging F-actin distribution, *UAS*-*Halo*-*Moesin* is also included. Complete fly genotypes of sparse axon experiments are described in Table S3. Flies were raised on standard cornmeal medium in a 12h/12h light cycle at 25°C (avoiding using 29°C to prevent any leakiness of *hsFLP*). Early-stage pupae (0–6 hours APF) were wrapped in a single layer of water-soaked paper towel (avoiding air bubbles to prevent inefficient heat transmission), heat shocked for 30 seconds in a 37°C water bath, and then immediately cooled for 60 seconds in a room temperature water bath (Figure S6B). Flies were dissected at 28–34, 34–40, 40–46 hours APF for Stage 1, Stage 2, and Stage 3, respectively. For fly stocks containing the sparse driver, *VT028327*-*FRT10*-*STOP*-*FRT10*-*p65AD* (or any other sparse drivers) and *hsFLP* are kept in separate stocks to avoid stochastic FLP expression and subsequent loss of the *FRT10*-*STOP*-*FRT10* cassette. Heat shock duration was empirically determined according to the intended number of cells, developmental stage, and tissue depth. If achieving the desired sparsity proves difficult, consider replacing the *FRT10*-*STOP*-*FRT10* element with a less sensitive *FRT100*-*STOP*-*FRT100* element^66^ in the sparse driver design.

### QUANTIFICATION AND STATISTICAL ANALYSIS

#### Quantification of match indices for DA1-ORNs

*Mz19*-*QF2*^*G4HACK*^-driven *QUAS*-*mtdTomato*-*3xHA* specifically labels DA1-PNs in most cases. Antennal lobes with occasional *Mz19*-*QF2*^*G4HACK*^-driven VA1d/DC3-PN labeling (cell bodies located dorsal to antennal lobe, rather than lateral for DA1-PNs) were excluded to prevent ambiguity in DA1-PN dendrite identification. DA1-ORN split GAL4–driven *UAS*-*mCD8*-*GFP* was used for DA1-ORN axon identification. “Match index” is defined as the ratio of the overlapping volume between DA1-ORN axons and DA1-PN dendrites to the total volume of DA1-PN dendrites. Data was analyzed using ImageJ (Fiji) 3D object counter and plotted using R. Data normality was assessed using the Shapiro-Wilk normality test. The Brown-Forsythe test was used to assess homoscedasticity prior to the ANOVA. For data with normal distribution and equal variance, the one-way ANOVA with Tukey’s test was used for multiple comparisons. Otherwise, the Kruskal-Wallis test with Bonferroni post-hoc correction was used for multiple comparisons.

#### Quantification of V5 signal intensities of Ten-m expression in DA1-ORNs

The average V5 signal intensity of each DA1 glomerulus was measured and then normalized against the maximum and minimum signal intensities within each image. Maximum signal intensities were primarily contributed by background signals from the trachea, whose intensity is consistent across fly brains. The Kruskal-Wallis test with Bonferroni post-hoc correction was used for multiple comparisons.

#### Quantification of mismatch indices in Mz19-PNs

*Mz19*-*GAL4*-driven *UAS*-*mCD8*-*GFP* was used for Mz19-PN dendrite identification, while *Or47b*-*rCD2* was used for VA1v-ORN axon identification. “Mismatch index” is defined as the ratio of the overlapping volume between VA1v-ORN axons and Mz19-PN dendrites to the total volume of VA1v-ORN axons. Data was analyzed using ImageJ (Fiji) 3D object counter and plotted using R. The Kruskal-Wallis test with Bonferroni post-hoc correction was used for multiple comparisons.

#### Quantification of mistarget indices in VA1d-ORNs

*Mz19*-*QF2*^*G4HACK*^-driven *QUAS*-*mtdTomato*-*3xHA* and the NCad staining were used to identify DA1 and VA1d glomeruli. VA1d-ORN split GAL4-driven *UAS*-*mCD8*-*GFP* was used for VA1d-ORN axon identification. “Mistarget index” is defined as the ratio of the total GFP fluorescence intensity of axons in the DA1 glomerulus to that in the DA1 and VA1d glomeruli. Data was analyzed using ImageJ (Fiji) and plotted using R. The Kruskal-Wallis test with Bonferroni post-hoc correction was used for multiple comparisons.

#### Image processing and quantification of sparse axon assays

Neurite tracing images were generated using Simple Neurite Tracer (SNT)^136^, processed using open-source R package natverse^137^, and analyzed and plotted in R. The stem axon was defined as the thickest segment of the axon. The antennal lobe entry point was determined by the first overlapping point of the axon (identified by GFP staining) and the antennal lobe (identified by NCad staining). The end point was defined as the farthest point of the stem axon from the antennal lobe entry point. The locations of primary branch points were normalized with the antennal lobe entry point set as 0 and the end point as 1. *Mz19*-*QF2*^*G4HACK*^-driven *QUAS*-*mtdTomato*-*3xHA* was used for DA1-PN dendrite identification. Branches extending to the DA1-PN dendrite region were categorized as “DA1-PN-contacting”. The chi-squared test with Bonferroni correction (Figure 6L and 6M) and the one-way ANOVA with Tukey’s test (Figure 6N–Q) were used for multiple comparisons.

Axons at stage 3 were used for F-actin analysis. Signal intensities of the F-actin marker (Halo-Moesin) along branches/segments, were traced using Simple Neurite Tracer, and quantified using ImageJ “Plot Profile” (integration metric: mean; sampling neighborhood: sphere with 1 pixel radius). Each node was normalized against the maximum and minimum signal intensities of each axon. For the comparison of F-actin density in whole branches (Figure 7D and 7H), F-actin densities were calculated by dividing the total normalized F-actin signal intensities of respective segments (whole branch here) by their lengths. The Mann-Whitney *U* test was used for comparisons. For the comparison of F-actin density in subbranches (Figure 7E), within primary branches that contact DA1-PN, segments with DA1-PN-contact were classified as “DA1-PN (+)”, and those without the contact as “DA1-PN (−)”. F-actin densities were calculated by dividing the total normalized F-actin signal intensities of respective segments (subbranch here) by their lengths. A paired *t* test was used for the comparison.

## Supplementary Material

1 **Table S2. Top candidates in the Ten-m intracellular interactome and their molecular features, related to** Figure 2 and Figure 3. Top 37 genes ranked by [APEX2-Ten-m/SR] fold change from the filtered Ten-m intracellular interactome (see Figure 3A red box). Human orthologs were identified using the FlyBase Homologs search tool, listing only those consistently recognized by four or more databases. Molecular features were referenced from FlyBase and UniProt.
Table S3. Complete genotypes of each experiment, related to STAR Methods.

2 **Figure S1. Characterization of Ten-m transgene expression, related to** Figure 1 (A–E) V5 staining in representative confocal images of antennal lobes of control at 29°C (A), *UAS*-*V5*-*Ten*-*m* overexpression at 25°C (B), *UAS*-*V5*-*Ten*-*m* overexpression at 29°C (C), *UAS*-*V5*-*Ten*-*m*-*ΔECD* overexpression at 29°C (D), and *UAS*-*V5*-*Ten*-*m*-*ΔICD* overexpression at 29°C (E). These are the same brains as shown in Figure 1F–H, 1K, and 1L, respectively.
(F) Quantification of normalized V5 intensities. V5 intensities were normalized to the maximum and minimum signal intensities of each image.
(G, G’) Representative confocal image of Ten-m overexpression in both DA1-ORN axons (green) and DA1-PN dendrites (magenta) (G), along with the image showing only DA1-PN dendrites and neuropil marker (G’).
(H) Match indices for (G) in comparison with two other experimental conditions (Figure 1I).
(I) Representative confocal images of full-length Ten-m (FL), Ten-m-ΔECD (ΔECD), or Ten-m-ΔICD (ΔICD) expressing S2 cells with plasma membrane permeabilized or non-permeabilized staining, respectively. Detection of the N-terminal V5 tag (magenta) exclusively in permeabilized staining, and the C-terminal FLAG tag (green) in both permeabilized and non-permeabilized conditions, consistent with expected protein localization and orientation on the plasma membrane (intracellular V5 and extracellular FLAG).
(J) Schematic of epitope tagging for constructs used in (I). All expression constructs are V5-tagged at the N-termini (magenta) and FLAG-tagged at the C-termini (green).
D, dorsal; L, lateral. Dashed white outline, antennal lobe. BRP, Bruchpilot, an active zone marker used for general neuropil staining. The Kruskal-Wallis test with Bonferroni post-hoc correction for multiple comparisons was used in (F) and (H). In this and all subsequent figures, * p < 0.05; ** p < 0.01; *** p < 0.001; n.s., not significant.

3 **Figure S2. Evidence that Ten-m-overexpressing DA1-ORNs mismatch with DL3-PNs, related to** Figure 1. (A) Tracings of DA1-ORNs and two types of DA1-PNs from the FlyWire dataset^82–84^ with relevant brain structures labeled. DA1-ORN axons from the left hemisphere (green) enter the antennal lobe ventrolaterally and innervate the left and right DA1 glomeruli of the antennal lobe. Excitatory DA1-PNs from the lateral neuroblast lineage (DA1-lPNs, purple) send dendrites to the DA1 glomeruli and axons through the inner antennocerebral tract (iACT) to innervate mushroom body calyx and lateral horn. A pair of GABAergic inhibitory DA1-PNs from the ventral neuroblast lineage (DA1-vPN, red) also send dendrites to the DA1 glomeruli and axons through the middle antennocerebral tract (mACT) to innervate only the lateral horn^138,139^.
(B, C) FlyWire tracings of DA1-lPNs and DA1-vPN from the left hemisphere (B), with a magnified view at the lateral horn (yellow box) to visualize their stereotyped axon branching patterns (C).
(D, E) Representative confocal images of *trans*-Tango-mediated trans-synaptic tracing from DA1-PNs of control. Green, ORN axons; magenta, postsynaptic neurons labeled by *trans*-Tango, which include dendrites of local interneurons and more intensely labeled DA1-PNs in the antennal lobe (D, D’) and DA1-PN axons in the lateral horn (E). Representative images from n = 6.
(F, G) FlyWire tracings of DA1-PNs (same as B, C) as well as DL3-PNs (cyan) (F), with a magnified view at the lateral horn (yellow box) to visualize their stereotyped axon branching patterns (G). Arrows indicate signature axon branches of DL3-PNs.
(H, I) Representative confocal images of *trans*-Tango-mediated trans-synaptic tracing from DA1-ORNs overexpressing Ten-m. Green, ORN axons; magenta, postsynaptic neurons labeled by *trans*-Tango, which includes not only local interneurons and DA1-PNs, but also notably dense labeling in the DL3 glomerulus (H, H’), as well as axons in the lateral horn (I) that is consistent with combined DA1-PN and DL3-PN axon pattern from FlyWire tracing (G). Arrows indicate signature axon branches of DL3-PNs (compared to panels E and G). Representative images from n = 8.
D, dorsal; L, lateral. Dashed white circle, antennal lobe. NCad, N-cadherin, a general neuropil marker.

4 **Figure S3. Analysis of unfiltered proteomes and the Ten-m intracellular interactome, related to** Figure 2. (A) Correlation of biological replicates. See Figure 2F for the assignment of the TMT labels.
(B) Top 18 protein domain terms (predicted by SMART) enriched in the Ten-m intracellular interactome.
(C) Top 14 reactome pathway terms enriched in the Ten-m intracellular interactome.
(D) Top 19 local network cluster terms enriched in the Ten-m intracellular interactome.

5 **Figure S4. Genetic interactions of Ten-m with Syd1, Cdc42, Rho1, and Gek in ORNs, related to** Figures 3 and 4. (A) Representative confocal images of DA1-PN dendrites (magenta) and DA1-ORN axons (green) of Ten-m-ΔICD overexpression with *Syd1*-*RNAi*.
(B) Match index of (A), which also includes Ten-m-ΔICD overexpression alone, as well as control and Ten-m overexpression data (from Figure 1M) for comparison.
(C–G) Representative confocal images of DA1-PN dendrites (magenta) and DA1-ORN axons (green) of *Cdc42*-*RNAi* (C), Ten-m overexpression with *Cdc42*-*RNAi* (D), *Rho1*-*RNAi* (E), and Ten-m overexpression with *Rho1*-*RNAi* (F). Match indices are quantified in (G), which also includes *Rac1*-*RNAi* (same data as in Figure 3S) for comparison as well as the control and Ten-m overexpression data from Figure 3I.
(H–L) Representative confocal images of DA1-PN dendrites (magenta) and DA1-ORN axons (green) of *Gek*-*RNAi* (H), Ten-m overexpression with *Gek*-*RNAi* (I), Gek overexpression (J), and Ten-m and Gek co-overexpression (K). Match indices are quantified in (L), which also includes the control and Ten-m overexpression data from Figure 3I.
(M) Protein domain organization of Gek. The asterisk (*) marks the lysine in the protein kinase domain essential for its catalytic activity.
(N) Co-immunoprecipitation of V5-tagged Ten-m and FLAG-tagged Gek proteins from co-transfected S2 cells. MW, molecular weight.
D, dorsal; L, lateral. Dashed white circle, antennal lobe. BRP, Bruchpilot, an active zone marker used for general neuropil staining. Mann-Whitney *U* test was used for the comparison (B). Kruskal-Wallis test with Bonferroni post-hoc correction for multiple comparisons was used in (G, L).

6 **Figure S5. Genetic interactions of Ten-m with Gek and Cdc42 in PNs, related to** Figure 4. (A–C) Representative confocal images of VA1v-ORN axons (magenta) and Mz19-PN dendrites (green) of *Gek*-*RNAi* (A) and Ten-m overexpression with *Gek*-*RNAi* (B). Mismatching indices are quantified in (C), which also includes the control and Ten-m overexpression data from Figure 4G.
(D–H) Representative confocal images of VA1v-ORN axons (magenta) and Mz19-PN dendrites (green) of Gek overexpression (D), Gek and Ten-m co-overexpression (E), Gek-K129A protein kinase-domain mutation overexpression (F), and Gek-K129A and Ten-m co-overexpression (G). Mismatch indices are quantified in (H), which also includes the control and Ten-m overexpression data from Figure 4G.
(I–M) Representative confocal images of VA1v-ORN axons (magenta) and Mz19-PN dendrites (green) of *Cdc42*-*RNAi* (I), Ten-m overexpression with *Cdc42*-*RNAi* (J), Cdc42 overexpression (K), and Cdc42 and Ten-m co-overexpression (L). Mismatching indices are quantified in (M), which also includes the control and Ten-m overexpression data from Figure 4G.
D, dorsal; L, lateral. Dashed white circle, antennal lobe. NCad, N-cadherin, a general neuropil marker. Arrowheads indicate overlap regions. Kruskal-Wallis test with Bonferroni post-hoc correction for multiple comparisons was used in (C), (H), and (M).

7 **Figure S6. The sparse driver strategy and single-axon analyses, related to** Figure 6. (A) The sparse driver strategy incorporates *hsFLP* (FLP recombinase driven by a heat-shock promoter), heat shock, and mutant *FRT* (*FRT10*) sites, where the A→T mutation (red) reduces recombination efficiency by 10-fold. Following recombination, the in-frame peptide derived from *FRT10* and *T2A* sequences is excised during the translation of the activation domain (AD).
(B) Protocol for activating the sparse driver in a single DA1-ORN.
(C, D) Representative maximum Z-projection images of a single DA1-ORN at Stage 2 (C) and Stage 3 (D).
(E–G) 3D trace Z-projections of the DA1-ORN axons of control at Stage 1 (E), Stage 2 (F), and Stage 3 (G).
(H–J) 3D trace Z-projections of the DA1-ORN axons of Ten-m overexpression at Stage 1 (H), Stage 2 (I), and Stage 3 (J).
(K–M) 3D trace Z-projections of the DA1-ORN axons of Ten-m overexpression with *Rac1*-*RNAi* at Stage 1 (K), Stage 2 (L), and Stage 3 (M).
D, dorsal; L, lateral. Orange square, axon entry point. Dark green, stem axon. Light green, axon branches. Yellow dot, primary branch point.

8 **Figure S7. Localizations of cytoskeleton markers in sparsely labeled developing ORN axons, related to** Figure 7. Representative maximum Z-projection images of sparse DA1-ORN axons with microtubule marker Halo-alphaTub84B (A and A’), microtubule plus-end marker Halo-EB1 (B and B’), or F-actin marker Halo-Moesin (C and C’). Arrowheads indicate signal peaks of differential distribution of the F-actin marker. D, dorsal; L, lateral. NCad, N-cadherin, a general neuropil marker.

9 **Table S1. Processed proteomic data, related to** Figure 2 (A) 3454 proteins detected in total.
(B) 2854 proteins with 2 or more unique peptides detected, corresponding to Figure 2I, Step 1.
(C) 781 proteins with [APEX2-Ten-m/NC] fold change higher than or equal to Ten-m’s [APEX2-Ten-m/NC] fold change, corresponding to Figure 2I, Step 2.
(D) 294 proteins with [APEX2-Ten-m/SR] fold change larger than 0, corresponding to Figure 2I, Step 3.

## Figures and Tables

**Table:** KEY RESOURCES TABLE

REAGENT or RESOURCE	SOURCE	IDENTIFIER
**Reagents**		
Schneider’s *Drosophila* medium	Thermo Fisher Scientific	Catalog #: 21720001
Fetal Bovine Serum, heat inactivated	Thermo Fisher Scientific	Catalog #: A3840101
Gateway LR Clonase II Enzyme Mix	Thermo Fisher Scientific	Catalog #: 11791020
pENTR/D-TOPO Cloning Kit	Thermo Fisher Scientific	Catalog #: K240020
Zero Blunt TOPO PCR Cloning Kit	Thermo Fisher Scientific	Catalog #: 450245
Phire Tissue Direct PCR Master Mix	Thermo Fisher Scientific	Catalog #: F170L
Q5 site-directed mutagenesis kit	New England Biolabs	Catalog #: E0554S
Q5 hot-start high-fidelity DNA polymerase	New England Biolabs	Catalog #: M0494S
NEBuilder HiFi DNA assembly master mix	New England Biolabs	Catalog #: E2621L
**Antibodies**		
rat anti-DNcad	Developmental Studies Hybridoma Bank	Catalog #: DN-Ex #8
mouse anti-BRP	Developmental Studies Hybridoma Bank	Catalog #: nc82
chicken anti-GFP	Aves Labs	Catalog #: GFP-1020
rabbit anti-DsRed	Clontech	Catalog #: 632496
mouse anti-rat CD2	Bio-Rad	Catalog #: OX-34
mouse anti-V5	Thermo Fisher Scientific	Catalog #: R960-25
rat anti-V5	Abcam	Catalog #: ab206571
Rabbit anti-HA	Cell Signaling	Catalog #: 3724S
mouse anti-FLAG	Sigma-Aldrich	Catalog #: F1804-200UG
**Experimental Models: Organisms/Strains**		
*D. melanogaster*: *GMR22E04-GAL4*^*DBD*^	(Jenett et al., 2012)	BDSC: 69199
*D. melanogaster*: *VT028327*-*p65*^*AD*^	(Tirian et al., 2017)	BDSC: 73064
*D. melanogaster*: *GMR31F09-GAL4*^*DBD*^	(Dionne et al., 2018)	BDSC: 68759
*D. melanogaster*: *GMR78H05*-*p65*^*AD*^	(Dionne et al., 2018)	BDSC: 70814
*D. melanogaster*: *Mz19*-*GAL4*	(Ito et al., 1998)	BDSC: 34497
*D. melanogaster*: *QUAS*-*mtdTomato-3xHA*	(Potter et al., 2010)	BDSC: 30004
*D. melanogaster*: *Or47b*-*rCD2*	(Zhu and Luo., 2004)	BDSC: 9916
*D. melanogaster*: *trans*-Tango	(Talay et al., 2017)	BDSC: 77123
*D. melanogaster*: *UAS*-*dcr2*	(Dietzl et al., 2007)	N/A
*D. melanogaster*: *UAS*-*mCD8*-*GFP*	(Lee and Luo., 1999)	DGRC: 108068
*D. melanogaster*: *hsFLP*	(Golic and Lindquist., 1989)	N/A
*D. melanogaster*: *NP*-*6658*-*GAL4*	(Hong et al., 2012)	BDSC: 41568
*D. melanogaster*: *P{GS}9267*	(Hong et al., 2012)	BDSC: 41567
*D. melanogaster*: *QUAS*-*Ten*-*m*	(Hong et al., 2012)	BDSC: 41571/41572
*D. melanogaster*: *UAS*-*myr-mGreenLantern*	(Wong et al., 2023)	N/A
*D. melanogaster*: *UAS*-*Syd1*-*WT-3xFLAG*	(Spinner et al., 2018)	N/A
*D. melanogaster*: *UAS*-*Syd1*-*R979A-3xFLAG*	(Spinner et al., 2018)	N/A
*D. melanogaster*: *UAS*-*Gek*	(Gontang et al., 2011)	N/A
*D. melanogaster*: *UAS*-*Gek*-*K129A*	(Gontang et al., 2011)	N/A
*D. melanogaster*: *Mz19*-*QF2*^*G4HACK*^	this study	N/A
*D. melanogaster*: *UAS*-*HaloalphaTub84B*	this study	N/A
*D. melanogaster*: *UAS*-*Halo*-*EB1*	this study	N/A
*D. melanogaster*: *UAS*-*Halo*-*Moesin*	this study	N/A
*D. melanogaster*: *UAS*-*V5*-*Ten*-*m*	this study	N/A
*D. melanogaster*: *UAS*-*V5*-*Ten*-*m*-*ΔECD*	this study	N/A
*D. melanogaster*: *UAS*-*V5*-*Ten*-*m*-*ΔICD*	this study	N/A
*D. melanogaster*: *APEX2*-*V5*-*Ten*-*m*	this study	N/A
*D. melanogaster*: *UAS*-*CD4*-*APEX2*	this study	N/A
*D. melanogaster*: *UAS*-*Gek*-*FLAG*	this study	N/A
*D. melanogaster*: *UAS*-*Gek*-*K129A-FLAG*	this study	N/A
*D. melanogaster*: *UAS*-*V5*-*Ten*-*m (RNAi*-*resistant)*	this study	N/A
*D. melanogaster*: *VT028327*-*FRT10-STOP*-*FRT10*-*p65AD*	this study	N/A
*D. melanogaster*: *UAS*-*RNAi* lines	(Dietzl et al., 2007; Ni et al., 2011; Perkins et al., 2015)	Stock numbers listed in Table S3
*D. melanogaster*: S2 cells	Thermo Fisher Scientific	Catalog #: R69007
**Recombinant DNA**		
*pUAS*-*FRT10*-*stop*-*FRT10*-*mCD8-GFP*	(Li et al., 2021)	N/A
*pCR*-*Blunt*-*TOPO*	Thermo Fisher Scientific	Catalog #: K280020
*pU6*-*BbsI*-*chiRNA*	(Gratz et al., 2014; Gratz et al., 2013)	Addgene: 45946
*UAS*-*Halo*-*CAAX*	(Sutcliffe et al., 2017)	Addgene: 87645
*pJFRC81*-*10xUAS*-*IVS*-*Syn21*-*GFP-p10*	(Pfeiffer et al., 2012)	Addgene: 36432
*UAS*-*CD4*-*GFP*	(Han et al., 2011)	N/A
*pUAST*-*attB*-*Ten*-*m*	(Hong et al., 2012)	N/A
Other tagged *UAS*-*Ten*-*m* constructs	this study	N/A
tagged *UAS*-*Gek* or -*Syd1* constructs	this study	N/A
*VT028327*-*FRT10*-*STOP*-*FRT10-p65AD* construct	this study	N/A
**Software and Algorithms**		
Zen	Carl Zeiss	RRID: SCR_013672
ImageJ	National Institutes of Health	RRID: SCR_003070
Illustrator	Adobe	RRID: SCR_010279
R	R Core Team	RRID:SCR_001905
RStudio	Posit, PBC	https://posit.co/
Spectrum Mill	Agilent	https://proteomics.broadinstitute.org/
**Others**		
Original mass Spectra data	MassIVE	MSV000094010

## HIGHLIGHTS

- *In situ* spatial proteomics reveals the intracellular interactome of teneurins
- Ten-m signals via a RhoGAP and Rac1 GTPase to regulate synaptic partner matching
- Single-axon analyses reveal a stabilization-upon-contact model for partner matching
- Ten-m signaling promotes F-actin in axon branches contacting partner dendrites
