# Supplementary material for: Molecular and Cellular Mechanisms of Teneurin Signaling in Synaptic Partner Matching
Source: Cell. Author manuscript; Available in PMC 2025 Feb 18. (PMC11833509; doi:10.1016/j.cell.2024.06.022)
Supplement: 1 — Table S2. Top candidates in the Ten-m intracellular interactome and their molecular features, related to Figure 2 and Figure 3. Top 37 genes ranked by [APEX2-Ten-m/SR] fold change from the filtered Ten-m intracellular interactome (see Figure 3A red box). Human orthologs were identified using the FlyBase Homologs search tool, listing only those consistently recognized by four or more databases. Molecular features were referenced from FlyBase and UniProt. Table S3. Complete genotypes of each experiment, related to STAR Methods. [file NIHMS2005581-supplement-1.pdf]

FlyBase ID	Gene	Molecular Feature	Human Ortholog	Plasma membrane	Cytoplasm	Endomembrane system	Synapse
FBgn0031500	*CG17221*	Predicted alcohol dehydrogenase [NAD(P)+] activity; involved in mushroom body development.	RTN4IP1	-	√	-	-
FBgn0025814	*Mgstl*	Predicted glutathione peroxidase activity and glutathione transferase activity	MGST1	-	√	-	-
FBgn0033540	*Elp2*	Elongator complex component; involved in tRNA modifications and STAT pathway regulation	ELP2	-	-	-	-
FBgn0003149	*Prm*	Unknown molecular function	-	-	√	-	-
FBgn0023519	*mRpL16*	Predicted rRNA binding activity; mitochondrial large ribosomal subunit	MRPL16	-	√	-	-
FBgn0035477	*CG14982*	Uncharacterized protein	FAM110A/B/C/D	-	-	-	-
FBgn0020272	*mst*	A Co-factor of the TCP-1 tubulin chaperone complex	MSTO1	-	√	-	-
FBgn0264574	*Glut1*	A transmembrane protein that transports glucose	SLC2A3	√	-	-	-
FBgn0004449	***Ten-m***	A type II transmembrane protein which instructs synaptic partner matching by homophillic attraction	TEN1-4	√	-	-	√
FBgn0085409	*smal*	A transmembrane protein detected in PN surface proteome and involved in wiring	DDR2/1	√	-	-	-
FBgn0260960	*Baldspot*	Fatty acid elongase activity	ELOVL6/3	-	√	√	-
FBgn0031815	*frj*	A lysophospholipid acyltransferase that preferentially adds arachidonic acid to lysophosphatidyli-sitol	MBOAT7/1/2/4	-	-	-	-
FBgn0001280	*janA*	Protein histidine phosphatase activity	PHPT1	-	√	-	-
FBgn0025726	*unc-13*	Involved in synaptic vesicle exocytosis; Calmodulin and syntaxin-1 binding	UNC13A-C	√	√	√	√
FBgn0037092	*M6*	A four-transmembrane glycoprotein involved in eye morphogenesis and oogenesis	GPM6A/B	√	-	-	-
FBgn0039883	***Syd1***	A GTPase activating protein for the Rho GTPases	SYDE1/2	-	-	-	√
FBgn0033313	*Cirl*	Predicted GPCR activity and latrotoxin receptor activity	ADGRL1-4	√	√	√	√
FBgn0023081	***Gek***	A Cdc42 downstream effector for the actin polymerization regulation	CDC42BPA/B/G	-	√	-	-
FBgn0032036	*CG13384*	Predicted L-amino acid transmembrane transporter activity	SLC36A1-4	-	-	-	-
FBgn0036821	*CG3961*	Predicted long-chain-fatty-acid-CoA ligase	ACSL1-6	-	√	√	-
FBgn0283724	*Girdin*	Predicted dynein light intermediate chain binding activity and microtubule binding activity	CCDC88A-C	-	√	-	-
FBgn0034657	*LBR*	Chromatin binding activity and lamin binding activity	LBR	-	-	√	-
FBgn0034590	*Magi*	Small GTPase binding activity	Magi1-3	√	√	-	√
FBgn0261649	*tinc*	A transmembrane protein involved in ommatidial cell development	-	√	-	-	-
FBgn0085478	*Zdhhc8*	A palmitoyltransferase that catalyzes the addition of palmitate onto various protein substrates	ZDHHC5/8	-	-	-	-
FBgn0025777	*homer*	A adaptor protein binding to group I mGluRs and other intracellular signaling proteins	HOMER1-3	√	√	√	-
FBgn0029175	*sotv*	Protein glycosylation and heparin synthesis	EXT2	-	√	√	-
FBgn0019925	*Surf4*	An ER cargo receptor for lipoprotein export via COPII vesicles.	SURF4	-	√	√	-
FBgn0039182	*CG5728*	Predicted RNA binding activity; involved in rRNA processing	PDCD11	-	-	-	-
FBgn0010434	*cora*	Predicted actin binding activity	EPB41L1-3	√	-	-	-
FBgn0020407	*asun*	Required for the correct dynein-dynactin perinuclear localization	INTS13	-	√	-	-
FBgn0031016	*kek5*	A transmembrane protein involved in BMP signaling regulation	LRFN1	√	-	-	-
FBgn0030930	*Pgant7*	Protein glycosylation	GALNT7	-	-	-	-
FBgn0031107	*HERC2*	Predicted ubiquitin protein ligase activity	HERC2	-	√	-	-
FBgn0039590	*CG10011*	Uncharacterized protein; Ankyrin repeat-containing domain superfamily	ANKRD50/23/28	-	-	-	-
FBgn0261671	*tweek*	Involved in synaptic vesicle endocytosis	BLTP1	-	-	-	√
FBgn0051163	*SKIP*	Unknown molecular function; SH3-like domain superfamily	SAMD5	-	-	-	-

Table S2. Top candidates in the Ten-m intracellular interactome and their molecular features, related to Figure 2 and Figure 3. Top 37 genes ranked by [APEX2-Ten-m/SR] fold change from the filtered Ten-m intracellular interactome (see Figure 3A red box). Human orthologs were identified using the FlyBase Homologs search tool, listing only those consistently recognized by four or more databases. Molecular features were referenced from FlyBase and UniProt.

Figure	Genotype
**Figure 1**	
C, F	UAS-dcr2, UAS-CD8-GFP / +; Mz19-QF2^{G4HACK} , QUAS-mtdTomato-3xHA, VT028327-p65^{AD} / +; GMR22E04-GAL4^{DBD} / +
D, G, H	UAS-dcr2, UAS-CD8-GFP / +; Mz19-QF2^{G4HACK} , QUAS-mtdTomato-3xHA, VT028327-p65^{AD} / +; GMR22E04-GAL4^{DBD}, UAS-V5-Ten-m / +
K	UAS-dcr2, UAS-CD8-GFP / +; Mz19-QF2^{G4HACK} , QUAS-mtdTomato-3xHA, VT028327-p65^{AD} / +; GMR22E04-GAL4^{DBD} / UAS-V5-Ten-m-ΔECD
L	UAS-dcr2, UAS-CD8-GFP / +; Mz19-QF2^{G4HACK} , QUAS-mtdTomato-3xHA, VT028327-p65^{AD} / +; GMR22E04-GAL4^{DBD} / UAS-V5-Ten-m-ΔICD
**Figure 2**	
C, D, E	APEX2-V5-Ten-m / APEX2-V5-Ten-m
F (126, 127)	Ten-m: APEX2-V5-Ten-m / APEX2-V5-Ten-m
F (128, 129)	SR: UAS-CD4-APEX2 / UAS-CD4-APEX2; NP-6658-GAL4 (Ten-m-GAL4) / NP-6658-GAL4 (Ten-m-GAL4)
F (130)	NC: wildtype (W1118)
F (131)	NC: APEX2-V5-Ten-m / APEX2-V5-Ten-m
**Figure 3**	
E	UAS-dcr2, UAS-CD8-GFP / +; Mz19-QF2^{G4HACK}, QUAS-mtdTomato-3xHA, VT028327-p65^{AD} / +; GMR22E04-GAL4^{DBD} / +
F	UAS-dcr2, UAS-CD8-GFP / +; Mz19-QF2^{G4HACK}, QUAS-mtdTomato-3xHA, VT028327-p65^{AD} / +; GMR22E04-GAL4^{DBD}, UAS-V5-Ten-m / +
G	UAS-dcr2, UAS-CD8-GFP / +; Mz19-QF2^{G4HACK}, QUAS-mtdTomato-3xHA, VT028327-p65^{AD} / UAS-Syd1-RNAi (BDRC_6446); GMR22E04-GAL4^{DBD} / +
H	UAS-dcr2, UAS-CD8-GFP / +; Mz19-QF2^{G4HACK}, QUAS-mtdTomato-3xHA, VT028327-p65^{AD} / UAS-Syd1-RNAi (BDRC_6446); GMR22E04-GAL4^{DBD}, UAS-V5-Ten-m / +
J	UAS-dcr2, UAS-CD8-GFP / +; Mz19-QF2^{G4HACK}, QUAS-mtdTomato-3xHA, VT028327-p65^{AD} / +; GMR22E04-GAL4^{DBD} / UAS-Syd1-WT-3xFLAG
K	UAS-dcr2, UAS-CD8-GFP / +; Mz19-QF2^{G4HACK}, QUAS-mtdTomato-3xHA, VT028327-p65^{AD} / +; GMR22E04-GAL4^{DBD}, UAS-V5-Ten-m / UAS-Syd1-WT-3xFLAG
L	UAS-dcr2, UAS-CD8-GFP / +; Mz19-QF2^{G4HACK}, QUAS-mtdTomato-3xHA, VT028327-p65^{AD} / +; GMR22E04-GAL4^{DBD} / UAS-Syd1-R979A-3xFLAG
M	UAS-dcr2, UAS-CD8-GFP / +; Mz19-QF2^{G4HACK}, QUAS-mtdTomato-3xHA, VT028327-p65^{AD} / +; GMR22E04-GAL4^{DBD}, UAS-V5-Ten-m / UAS-Syd1-979A-3xFLAG
O	UAS-dcr2, UAS-CD8-GFP / +; Mz19-QF2^{G4HACK}, QUAS-mtdTomato-3xHA, VT028327-p65^{AD} / +; GMR22E04-GAL4^{DBD} / UAS-Rac1-RNAi (BDRC_28985)
P	UAS-dcr2, UAS-CD8-GFP / +; Mz19-QF2^{G4HACK}, QUAS-mtdTomato-3xHA, VT028327-p65^{AD} / +; GMR22E04-GAL4^{DBD}, UAS-V5-Ten-m / UAS-Rac1-RNAi (BDRC_28985)
Q	UAS-dcr2, UAS-CD8-GFP / +; Mz19-QF2^{G4HACK}, QUAS-mtdTomato-3xHA, VT028327-p65^{AD} / +; GMR22E04-GAL4^{DBD} / UAS-Rac1 (BDRC_28874)
R	UAS-dcr2, UAS-CD8-GFP / +; Mz19-QF2^{G4HACK}, QUAS-mtdTomato-3xHA, VT028327-p65^{AD} / +; GMR22E04-GAL4^{DBD}, UAS-V5-Ten-m / UAS-Rac1 (BDRC_28874)
**Figure 4**	
B	UAS-dcr2, UAS-CD8-GFP / +; Mz19-GAL4, UAS-CD8-GFP / +; Or47b-rCD2 / +
C	UAS-dcr2, UAS-CD8-GFP / +; Mz19-GAL4, UAS-CD8-GFP / +; Or47b-rCD2, P{GS}9267 (UAS-gated Ten-m overexpression) / +
D	UAS-dcr2, UAS-CD8-GFP / +; Mz19-GAL4, UAS-CD8-GFP / UAS-Syd1-RNAi (BDRC_6446); Or47b-rCD2 / +
E	UAS-dcr2, UAS-CD8-GFP / +; Mz19-GAL4, UAS-CD8-GFP / UAS-Syd1-RNAi (BDRC_6446); Or47b-rCD2, P{GS}9267 (UAS-gated Ten-m overexpression) / +
H	UAS-dcr2, UAS-CD8-GFP / +; Mz19-GAL4, UAS-CD8-GFP / +; Or47b-rCD2 / UAS-Syd1-WT-3xFLAG
I	UAS-dcr2, UAS-CD8-GFP / +; Mz19-GAL4, UAS-CD8-GFP / +; Or47b-rCD2, P{GS}9267 (UAS-gated Ten-m overexpression) / UAS-Syd1-WT-3xFLAG
J	UAS-dcr2, UAS-CD8-GFP / +; Mz19-GAL4, UAS-CD8-GFP / +; Or47b-rCD2 / UAS-Syd1-R979A-3xFLAG
K	UAS-dcr2, UAS-CD8-GFP / +; Mz19-GAL4, UAS-CD8-GFP / +; Or47b-rCD2, P{GS}9267 (UAS-gated Ten-m overexpression) / UAS-Syd1-R979A-3xFLAG
M	UAS-dcr2, UAS-CD8-GFP / +; Mz19-GAL4, UAS-CD8-GFP / +; Or47b-rCD2 / UAS-Rac1-RNAi (BDRC_28985)
N	UAS-dcr2, UAS-CD8-GFP / +; Mz19-GAL4, UAS-CD8-GFP / +; Or47b-rCD2, P{GS}9267 (UAS-gated Ten-m overexpression) / UAS-Rac1-RNAi (BDRC_28985)
O	UAS-dcr2, UAS-CD8-GFP / +; Mz19-GAL4, UAS-CD8-GFP / +; Or47b-rCD2 / UAS-Rac1 (BDRC_28874)
P	UAS-dcr2, UAS-CD8-GFP / +; Mz19-GAL4, UAS-CD8-GFP / +; Or47b-rCD2, P{GS}9267 (UAS-gated Ten-m overexpression) / UAS-Rac1 (BDRC_28874)
**Figure 5**	
B	UAS-dcr2, UAS-CD8-GFP / +; Mz19-QF2^{G4HACK}, QUAS-mtdTomato-3xHA, GMR78H05-p65^{AD} / +; GMR31F09-GAL4^{DBD} / +
C, D	UAS-dcr2, UAS-CD8-GFP / +; Mz19-QF2^{G4HACK}, QUAS-mtdTomato-3xHA, GMR78H05-p65^{AD} / UAS-Ten-m-RNAi (VDRC_330540); GMR31F09-GAL4^{DBD} / +
E	UAS-dcr2, UAS-CD8-GFP / +; Mz19-QF2^{G4HACK}, QUAS-mtdTomato-3xHA, GMR78H05-p65^{AD} / UAS-Ten-m-RNAi (VDRC_330540); GMR31F09-GAL4^{DBD} / UAS-V5-Ten-m (RNAi-resistant)
I	UAS-dcr2, UAS-CD8-GFP / +; Mz19-QF2^{G4HACK}, QUAS-mtdTomato-3xHA, GMR78H05-p65^{AD} / UAS-Syd1-RNAi (BDRC_6446); GMR31F09-GAL4^{DBD} / +
J	UAS-dcr2, UAS-CD8-GFP / +; Mz19-QF2^{G4HACK}, QUAS-mtdTomato-3xHA, GMR78H05-p65^{AD} / UAS-Syd1-RNAi (BDRC_6446), UAS-Ten-m-RNAi (VDRC_330540); GMR31F09-GAL4^{DBD} / +
K	UAS-dcr2, UAS-CD8-GFP / +; Mz19-QF2^{G4HACK}, QUAS-mtdTomato-3xHA, GMR78H05-p65^{AD} / +; GMR31F09-GAL4^{DBD} / UAS-Rac1 (BDRC_28874)
L	UAS-dcr2, UAS-CD8-GFP / +; Mz19-QF2^{G4HACK}, QUAS-mtdTomato-3xHA, GMR78H05-p65^{AD} / UAS-Ten-m-RNAi (VDRC_330540); GMR31F09-GAL4^{DBD} / UAS-Rac1 (BDRC_28874)
**Figure 6**	
B (top)	UAS-dcr2, UAS-CD8-GFP / +; VT028327-p65^{AD} / +; GMR22E04-GAL4$^{DBD /}$ +
B (others)	UAS-CD8-GFP, hsFLP / UAS-dcr2, UAS-CD8-GFP; GMR22E04-GAL4^{DBD} / VT028327FRT10-STOP-FRT10-p65^{AD}
C, F'—H'	UAS-CD8-GFP, hsFLP / UAS-dcr2, UAS-CD8-GFP; Mz19-QF2^{G4HACK}, QUAS-mtdTomato-3xHA / UAS-myr-mGreenLantern; GMR22E04-GAL4^{DBD} / VT028327FRT10-STOP-FRT10-p65^{AD}
F"—H"	UAS-CD8-GFP, hsFLP / UAS-dcr2, UAS-CD8-GFP; Mz19-QF2^{G4HACK}, QUAS-mtdTomato-3xHA / UAS-myr-mGreenLantern; GMR22E04-GAL4^{DBD}, UAS-V5-Ten-m / VT028327FRT10-STOP-FRT10-p65^{AD}
F'''—H'''	UAS-CD8-GFP, hsFLP / UAS-dcr2, UAS-CD8-GFP; Mz19-QF2^{G4HACK}, QUAS-mtdTomato-3xHA / UAS-myr-mGreenLantern; GMR22E04-GAL4^{DBD}, UAS-V5-Ten-m / VT028327FRT10-STOP-FRT10-p65^{AD}, UAS-Rac1-RNAi (BDRC_28985)

**Figure 7**	
**A**	UAS-CD8-GFP, hsFLP / UAS-dcr2, UAS-CD8-GFP; Mz19-QF2^{G4HACK}, QUAS-mtdTomato-3xHA / UAS-myr-mGreenLantern; GMR22E04-GAL4^{DBD} / VT028327FRT10-STOP-FRT10-p65^{AD}, UAS-Halo-Moesin
**B**	UAS-CD8-GFP, hsFLP / UAS-dcr2, UAS-CD8-GFP; Mz19-QF2^{G4HACK}, QUAS-mtdTomato-3xHA / UAS-myr-mGreenLantern; GMR22E04-GAL4^{DBD}, UAS-V5-Ten-m / VT028327FRT10-STOP-FRT10-p65^{AD}, UAS-Halo-Moesin
**C**	UAS-CD8-GFP, hsFLP / UAS-dcr2, UAS-CD8-GFP; Mz19-QF2^{G4HACK}, QUAS-mtdTomato-3xHA / UAS-myr-mGreenLantern; GMR22E04-GAL4^{DBD}, UAS-V5-Ten-m / VT028327FRT10-STOP-FRT10-p65^{AD}, UAS-Rac1-RNAi (BDRC_28985), UAS-Halo-Moesin
**Figure S1**	
**A**	UAS-dcr2, UAS-CD8-GFP / +; Mz19-QF2^{G4HACK} , QUAS-mtdTomato-3xHA, VT028327-p65^{AD} / +; GMR22E04-GAL4^{DBD} / +
**B, C**	UAS-dcr2, UAS-CD8-GFP / +; Mz19-QF2^{G4HACK} , QUAS-mtdTomato-3xHA, VT028327-p65^{AD} / +; GMR22E04-GAL4^{DBD}, UAS-V5-Ten-m / +
**D**	UAS-dcr2, UAS-CD8-GFP / +; Mz19-QF2^{G4HACK} , QUAS-mtdTomato-3xHA, VT028327-p65^{AD} / +; GMR22E04-GAL4^{DBD} / UAS-V5-Ten-m-ΔECD
**E**	UAS-dcr2, UAS-CD8-GFP / +; Mz19-QF2^{G4HACK} , QUAS-mtdTomato-3xHA, VT028327-p65^{AD} / +; GMR22E04-GAL4^{DBD} / UAS-V5-Ten-m-ΔICD
**G**	UAS-dcr2, UAS-CD8-GFP / +; Mz19-QF2^{G4HACK} , QUAS-mtdTomato-3xHA, VT028327-p65^{AD} / QUAS-Ten-m; GMR22E04-GAL4^{DBD}, UAS-V5-Ten-m / QUAS-Ten-m
**Figure S2**	
**D, E**	no heat shock: QUAS-mtdTomato-3xHA, UAS-CD8-GFP, hsFLP / + ; trans-TANGO / +; GMR22E04-GAL4^{DBD}, UAS-V5-Ten-m / VT028327FRT10-STOP-FRT10-p65^{AD}
**H, I**	heat shock: QUAS-mtdTomato-3xHA, UAS-CD8-GFP, hsFLP / + ; trans-TANGO / +; GMR22E04-GAL4^{DBD}, UAS-V5-Ten-m / VT028327FRT10-STOP-FRT10-p65^{AD}
**Figure S4**	
**A**	UAS-dcr2, UAS-CD8-GFP / +; Mz19-QF2^{G4HACK} , QUAS-mtdTomato-3xHA, VT028327-p65^{AD} / UAS-Syd1-RNAi (BDRC_6446); GMR22E04-GAL4^{DBD} / UAS-V5-Ten-m-ΔICD
**C**	UAS-dcr2, UAS-CD8-GFP / +; Mz19-QF2^{G4HACK}, QUAS-mtdTomato-3xHA, VT028327-p65^{AD} / UAS-Cdc42-RNAi (BDRC_37477); GMR22E04-GAL4^{DBD} / +
**D**	UAS-dcr2, UAS-CD8-GFP / +; Mz19-QF2^{G4HACK}, QUAS-mtdTomato-3xHA, VT028327-p65^{AD} / UAS-Cdc42-RNAi (BDRC_37477); GMR22E04-GAL4^{DBD}, UAS-V5-Ten-m / +
**E**	UAS-dcr2, UAS-CD8-GFP / +; Mz19-QF2^{G4HACK}, QUAS-mtdTomato-3xHA, VT028327-p65^{AD} / UAS-Rho1-RNAi (BDRC_29003); GMR22E04-GAL4^{DBD} / +
**F**	UAS-dcr2, UAS-CD8-GFP / +; Mz19-QF2^{G4HACK}, QUAS-mtdTomato-3xHA, VT028327-p65^{AD} / UAS-Rho1-RNAi (BDRC_29003); GMR22E04-GAL4^{DBD}, UAS-V5-Ten-m / +
**H**	UAS-dcr2, UAS-CD8-GFP / +; Mz19-QF2^{G4HACK}, QUAS-mtdTomato-3xHA, VT028327-p65^{AD} / UAS-Gek-RNAi (BDRC_57379); GMR22E04-GAL4^{DBD} / +
**I**	UAS-dcr2, UAS-CD8-GFP / +; Mz19-QF2^{G4HACK}, QUAS-mtdTomato-3xHA, VT028327-p65^{AD} / UAS-Gek-RNAi (BDRC_57379); GMR22E04-GAL4^{DBD}, UAS-V5-Ten-m / +
**J**	UAS-dcr2, UAS-CD8-GFP / +; Mz19-QF2^{G4HACK}, QUAS-mtdTomato-3xHA, VT028327-p65^{AD} / UAS-Gek-FLAG; GMR22E04-GAL4^{DBD} / +
**K**	UAS-dcr2, UAS-CD8-GFP / +; Mz19-QF2^{G4HACK}, QUAS-mtdTomato-3xHA, VT028327-p65^{AD} / UAS-Gek-FLAG; GMR22E04-GAL4^{DBD}, UAS-V5-Ten-m / +
**Figure S5**	
**A**	UAS-dcr2, UAS-CD8-GFP / +; Mz19-GAL4, UAS-CD8-GFP / UAS-Gek-RNAi (BDRC_57379); Or47b-rCD2 / +
**B**	UAS-dcr2, UAS-CD8-GFP / +; Mz19-GAL4, UAS-CD8-GFP / UAS-Gek-RNAi (BDRC_57379); Or47b-rCD2, P{GS}9267 (UAS-gated Ten-m overexpression) / +
**D**	UAS-dcr2, UAS-CD8-GFP / +; Mz19-GAL4, UAS-CD8-GFP / UAS-Gek-FLAG; Or47b-rCD2 / +
**E**	UAS-dcr2, UAS-CD8-GFP / +; Mz19-GAL4, UAS-CD8-GFP / UAS-Gek-FLAG; Or47b-rCD2, P{GS}9267 (UAS-gated Ten-m overexpression) / +
**F**	UAS-dcr2, UAS-CD8-GFP / +; Mz19-GAL4, UAS-CD8-GFP / UAS-Gek-K129A-FLAG; Or47b-rCD2 / +
**G**	UAS-dcr2, UAS-CD8-GFP / +; Mz19-GAL4, UAS-CD8-GFP / UAS-Gek-K129A-FLAG; Or47b-rCD2, P{GS}9267 (UAS-gated Ten-m overexpression) / +
**I**	UAS-dcr2, UAS-CD8-GFP / +; Mz19-GAL4, UAS-CD8-GFP / UAS-Cdc42-RNAi (BDRC_37477); Or47b-rCD2 / +
**J**	UAS-dcr2, UAS-CD8-GFP / +; Mz19-GAL4, UAS-CD8-GFP / UAS-Cdc42-RNAi (BDRC_37477); Or47b-rCD2, P{GS}9267 (UAS-gated Ten-m overexpression) / +
**K**	UAS-dcr2, UAS-CD8-GFP / +; Mz19-GAL4, UAS-CD8-GFP / +; Or47b-rCD2 / UAS-Cdc42 (BDRC_28873)
**L**	UAS-dcr2, UAS-CD8-GFP / +; Mz19-GAL4, UAS-CD8-GFP / +; Or47b-rCD2, P{GS}9267 (UAS-gated Ten-m overexpression) / UAS-Cdc42 (BDRC_28873)
**Figure S6**	
**C, D, E, F, G**	UAS-CD8-GFP, hsFLP / UAS-dcr2, UAS-CD8-GFP; Mz19-QF2^{G4HACK}, QUAS-mtdTomato-3xHA / UAS-myr-mGreenLantern; GMR22E04-GAL4^{DBD} / VT028327FRT10-STOP-FRT10-p65^{AD}
**H, I, J**	UAS-CD8-GFP, hsFLP / UAS-dcr2, UAS-CD8-GFP; Mz19-QF2^{G4HACK}, QUAS-mtdTomato-3xHA / UAS-myr-mGreenLantern; GMR22E04-GAL4^{DBD}, UAS-V5-Ten-m / VT028327FRT10-STOP-FRT10-p65^{AD}
**K, L, M**	UAS-CD8-GFP, hsFLP / UAS-dcr2, UAS-CD8-GFP; Mz19-QF2^{G4HACK}, QUAS-mtdTomato-3xHA / UAS-myr-mGreenLantern; GMR22E04-GAL4^{DBD}, UAS-V5-Ten-m / VT028327FRT10-STOP-FRT10-p65^{AD}, UAS-Rac1-RNAi (BDRC_28985)
**Figure S7**	
**A**	UAS-CD8-GFP, hsFLP / UAS-dcr2, UAS-CD8-GFP; Mz19-QF2^{G4HACK}, QUAS-mtdTomato-3xHA / UAS-myr-mGreenLantern; GMR22E04-GAL4^{DBD} / VT028327FRT10-STOP-FRT10-p65^{AD}, UAS-Halo-alphaTub84B
**B**	UAS-CD8-GFP, hsFLP / UAS-dcr2, UAS-CD8-GFP; Mz19-QF2^{G4HACK}, QUAS-mtdTomato-3xHA / UAS-myr-mGreenLantern; GMR22E04-GAL4^{DBD} / VT028327FRT10-STOP-FRT10-p65^{AD}, UAS-Halo-EB1
**C**	UAS-CD8-GFP, hsFLP / UAS-dcr2, UAS-CD8-GFP; Mz19-QF2^{G4HACK}, QUAS-mtdTomato-3xHA / UAS-myr-mGreenLantern; GMR22E04-GAL4^{DBD} / VT028327FRT10-STOP-FRT10-p65^{AD}, UAS-Halo-Moesin

Table S3. Complete genotypes of each experiment, related to STAR Methods.
