# Supplementary figures and images for: Molecular and Cellular Mechanisms of Teneurin Signaling in Synaptic Partner Matching
Source: Cell. Author manuscript; Available in PMC 2025 Feb 18. (PMC11833509; doi:10.1016/j.cell.2024.06.022)

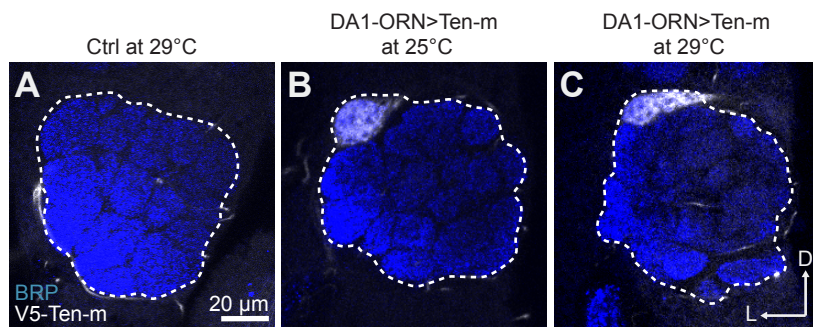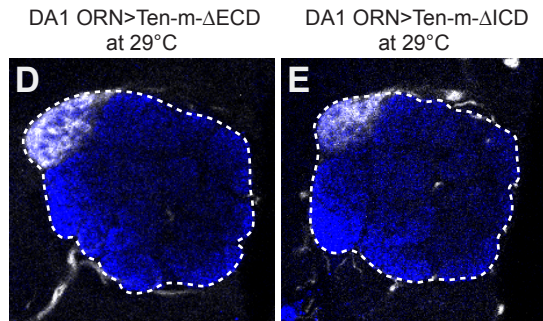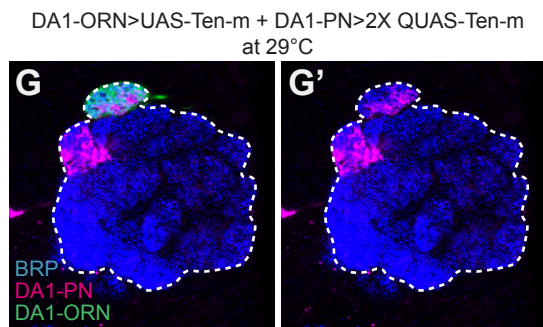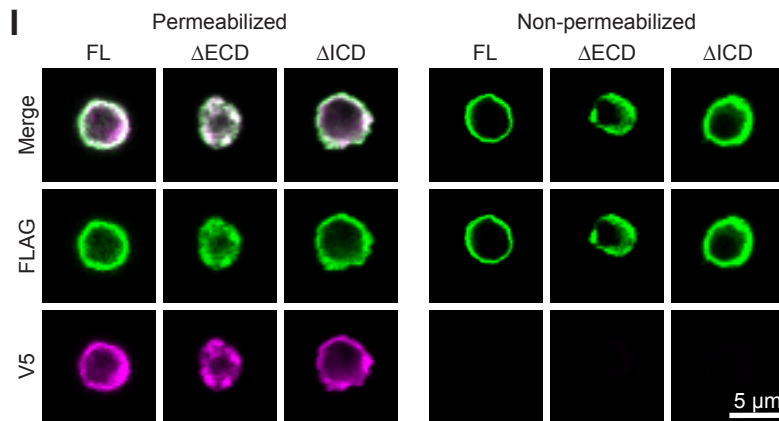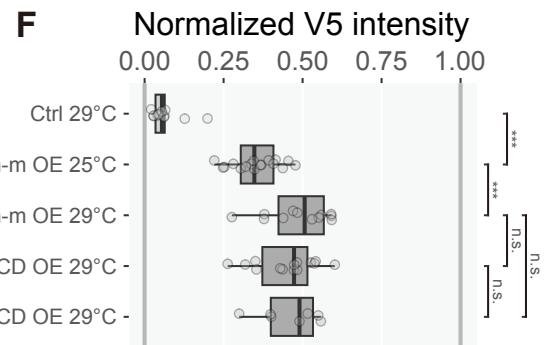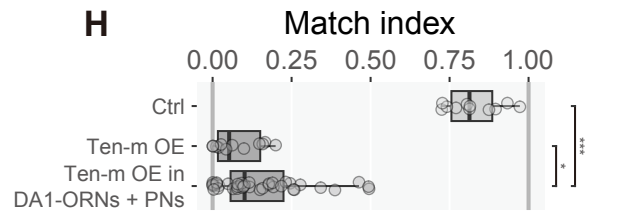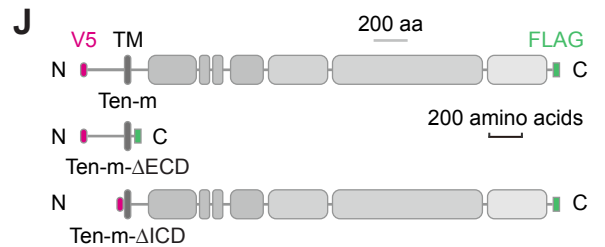

Supplement: 2 — Figure S1. Characterization of Ten-m transgene expression, related to Figure 1 (A–E) V5 staining in representative confocal images of antennal lobes of control at 29°C (A), UAS-V5-Ten-m overexpression at 25°C (B), UAS-V5-Ten-m overexpression at 29°C (C), UAS-V5-Ten-m-ΔECD overexpression at 29°C (D), and UAS-V5-Ten-m-ΔICD overexpression at 29°C (E). These are the same brains as shown in Figure 1F–H, 1K, and 1L, respectively. (F) Quantification of normalized V5 intensities. V5 intensities were normalized to the maximum and minimum signal intensities of each image. (G, G’) Representative confocal image of Ten-m overexpression in both DA1-ORN axons (green) and DA1-PN dendrites (magenta) (G), along with the image showing only DA1-PN dendrites and neuropil marker (G’). (H) Match indices for (G) in comparison with two other experimental conditions (Figure 1I). (I) Representative confocal images of full-length Ten-m (FL), Ten-m-ΔECD (ΔECD), or Ten-m-ΔICD (ΔICD) expressing S2 cells with plasma membrane permeabilized or non-permeabilized staining, respectively. Detection of the N-terminal V5 tag (magenta) exclusively in permeabilized staining, and the C-terminal FLAG tag (green) in both permeabilized and non-permeabilized conditions, consistent with expected protein localization and orientation on the plasma membrane (intracellular V5 and extracellular FLAG). (J) Schematic of epitope tagging for constructs used in (I). All expression constructs are V5-tagged at the N-termini (magenta) and FLAG-tagged at the C-termini (green). D, dorsal; L, lateral. Dashed white outline, antennal lobe. BRP, Bruchpilot, an active zone marker used for general neuropil staining. The Kruskal-Wallis test with Bonferroni post-hoc correction for multiple comparisons was used in (F) and (H). In this and all subsequent figures, * p < 0.05; ** p < 0.01; *** p < 0.001; n.s., not significant. [file NIHMS2005581-supplement-2.pdf]

**A**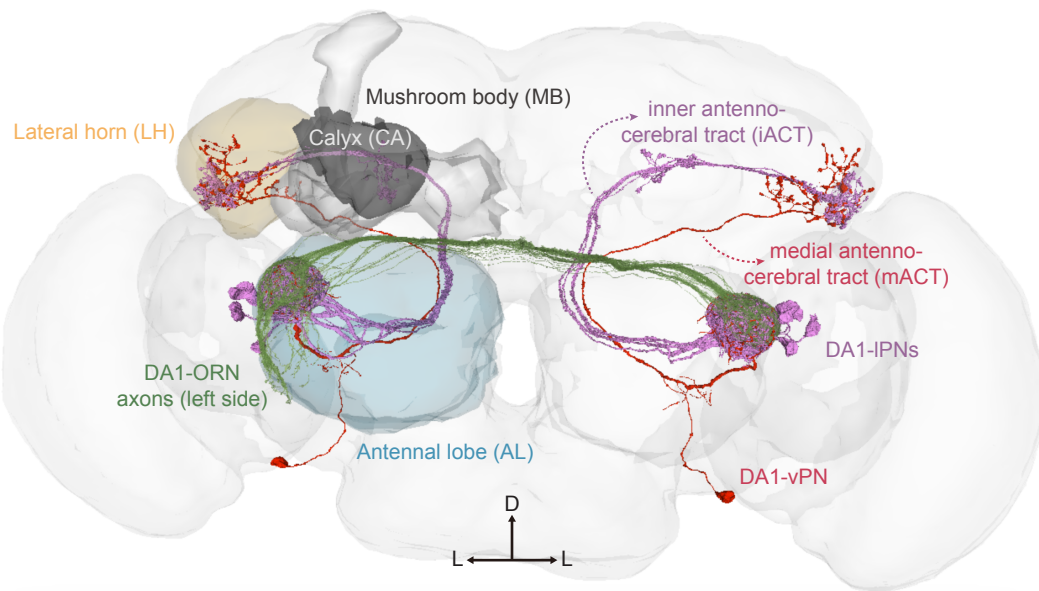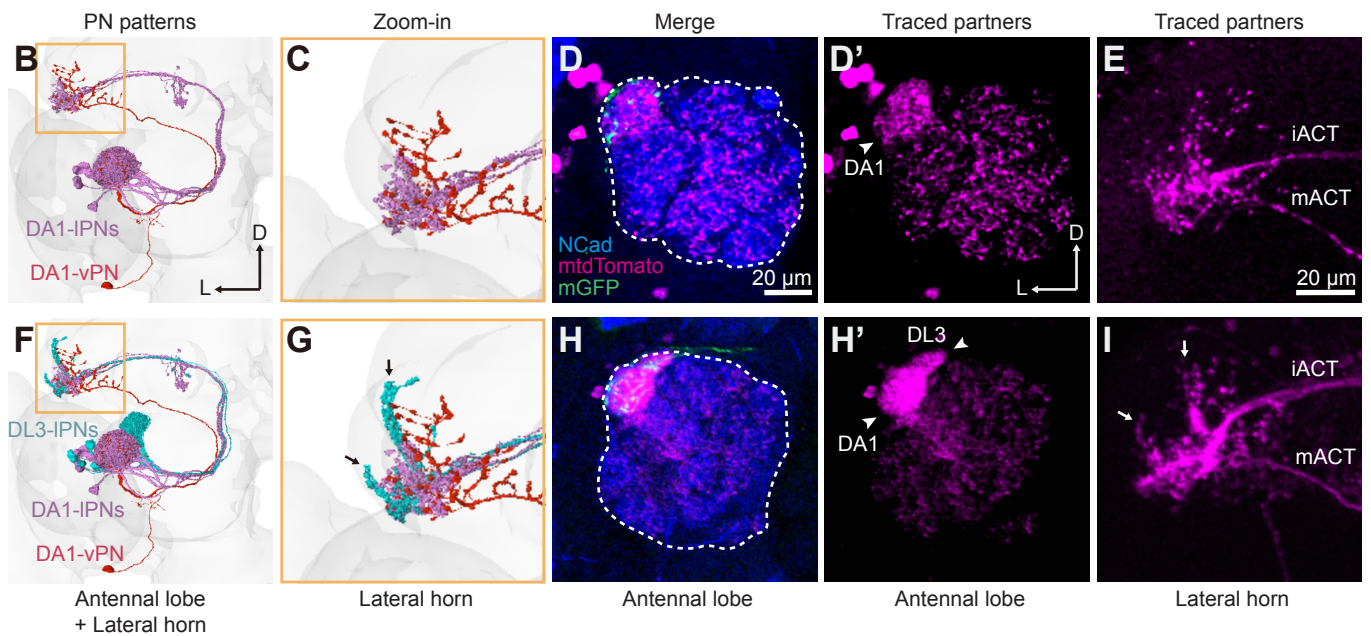

Supplement: 3 — Figure S2. Evidence that Ten-m-overexpressing DA1-ORNs mismatch with DL3-PNs, related to Figure 1. (A) Tracings of DA1-ORNs and two types of DA1-PNs from the FlyWire dataset82–84 with relevant brain structures labeled. DA1-ORN axons from the left hemisphere (green) enter the antennal lobe ventrolaterally and innervate the left and right DA1 glomeruli of the antennal lobe. Excitatory DA1-PNs from the lateral neuroblast lineage (DA1-lPNs, purple) send dendrites to the DA1 glomeruli and axons through the inner antennocerebral tract (iACT) to innervate mushroom body calyx and lateral horn. A pair of GABAergic inhibitory DA1-PNs from the ventral neuroblast lineage (DA1-vPN, red) also send dendrites to the DA1 glomeruli and axons through the middle antennocerebral tract (mACT) to innervate only the lateral horn138,139. (B, C) FlyWire tracings of DA1-lPNs and DA1-vPN from the left hemisphere (B), with a magnified view at the lateral horn (yellow box) to visualize their stereotyped axon branching patterns (C). (D, E) Representative confocal images of trans-Tango-mediated trans-synaptic tracing from DA1-PNs of control. Green, ORN axons; magenta, postsynaptic neurons labeled by trans-Tango, which include dendrites of local interneurons and more intensely labeled DA1-PNs in the antennal lobe (D, D’) and DA1-PN axons in the lateral horn (E). Representative images from n = 6. (F, G) FlyWire tracings of DA1-PNs (same as B, C) as well as DL3-PNs (cyan) (F), with a magnified view at the lateral horn (yellow box) to visualize their stereotyped axon branching patterns (G). Arrows indicate signature axon branches of DL3-PNs. (H, I) Representative confocal images of trans-Tango-mediated trans-synaptic tracing from DA1-ORNs overexpressing Ten-m. Green, ORN axons; magenta, postsynaptic neurons labeled by trans-Tango, which includes not only local interneurons and DA1-PNs, but also notably dense labeling in the DL3 glomerulus (H, H’), as well as axons in the lateral horn (I) that is consi [file NIHMS2005581-supplement-3.pdf]

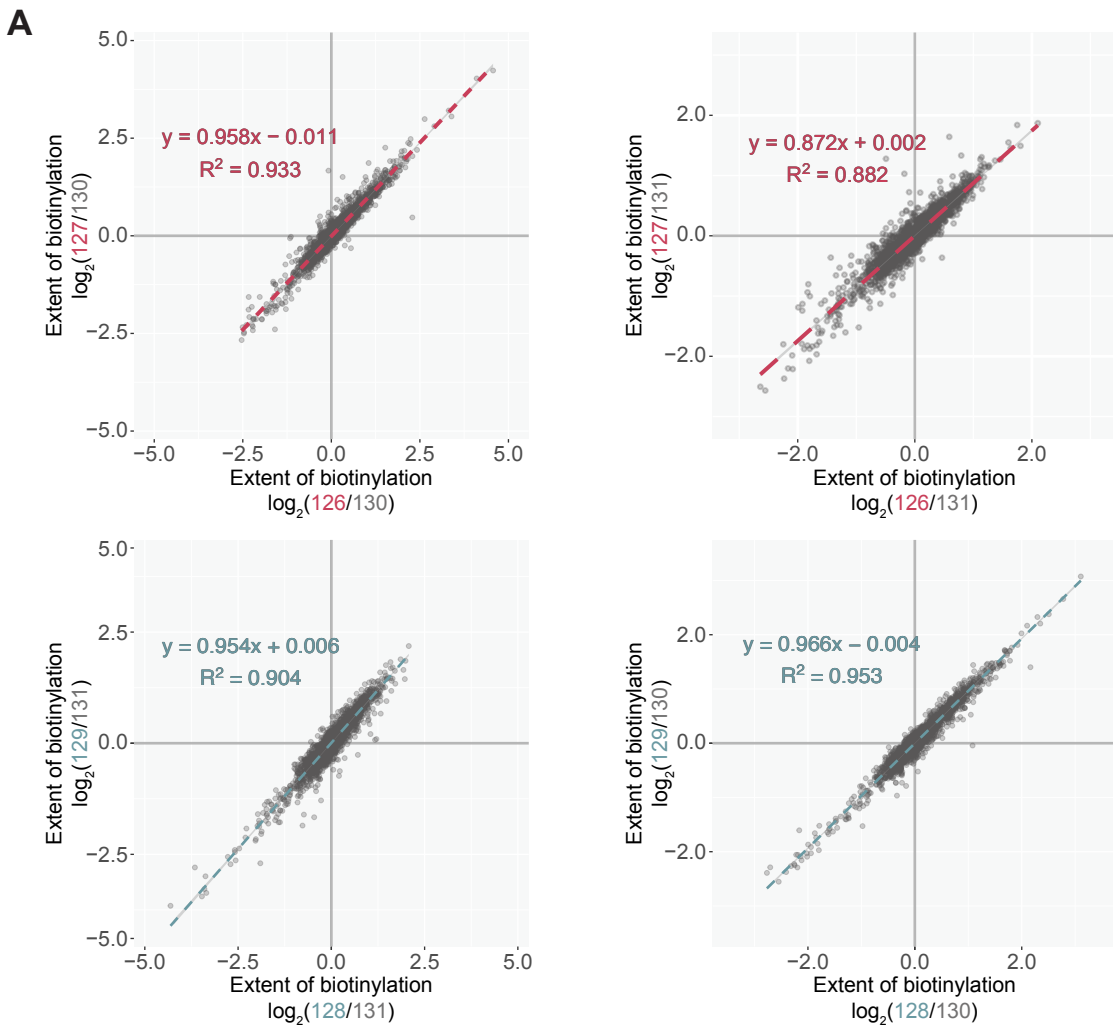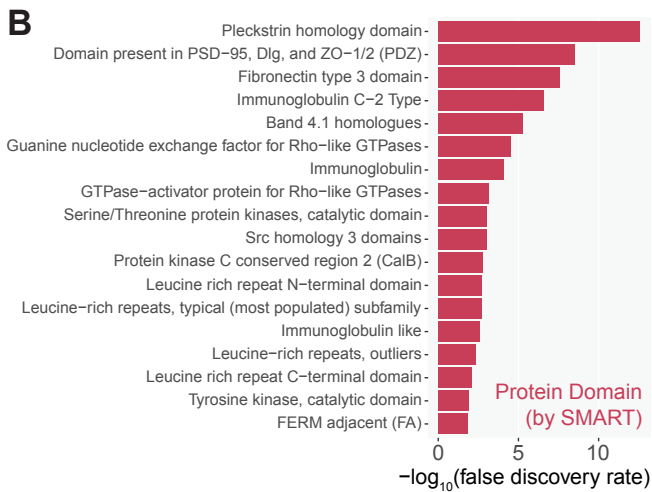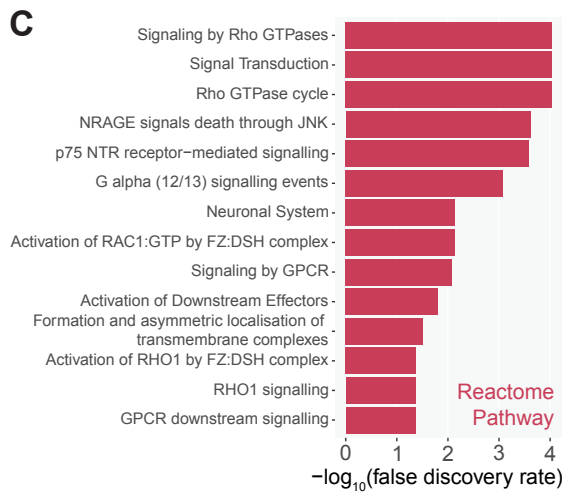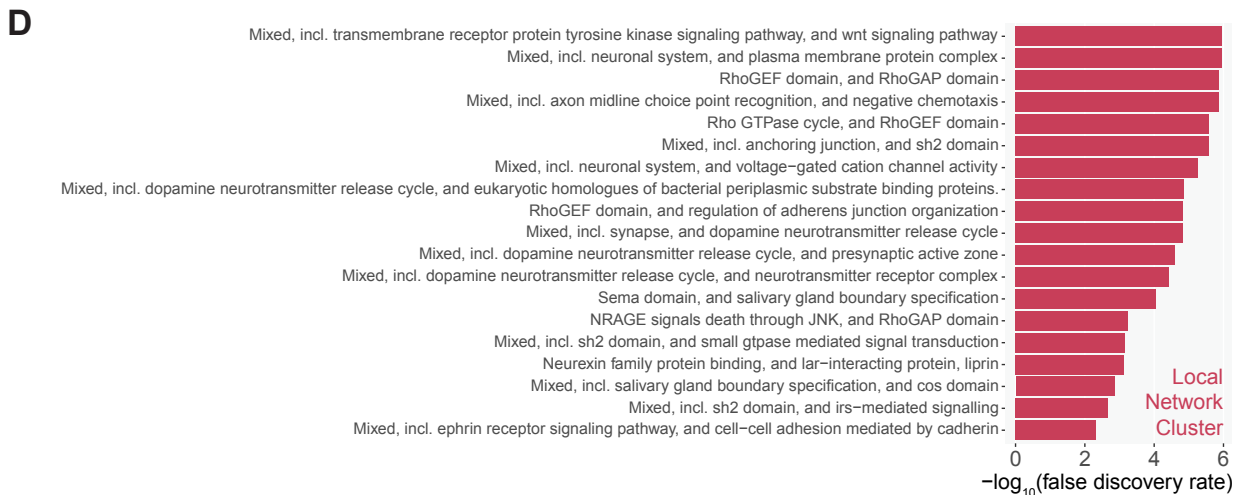

Supplement: 4 — Figure S3. Analysis of unfiltered proteomes and the Ten-m intracellular interactome, related to Figure 2. (A) Correlation of biological replicates. See Figure 2F for the assignment of the TMT labels. (B) Top 18 protein domain terms (predicted by SMART) enriched in the Ten-m intracellular interactome. (C) Top 14 reactome pathway terms enriched in the Ten-m intracellular interactome. (D) Top 19 local network cluster terms enriched in the Ten-m intracellular interactome. [file NIHMS2005581-supplement-4.pdf]

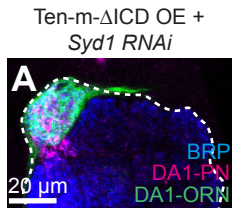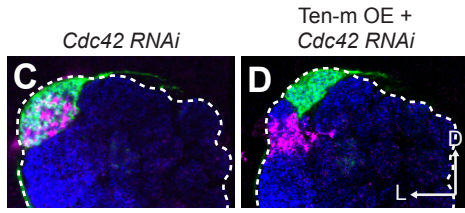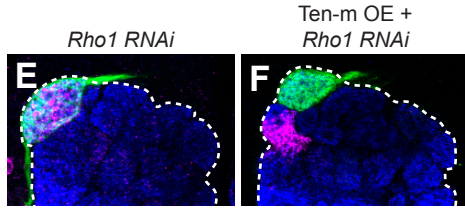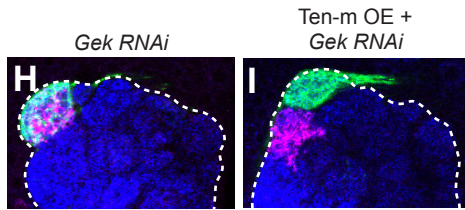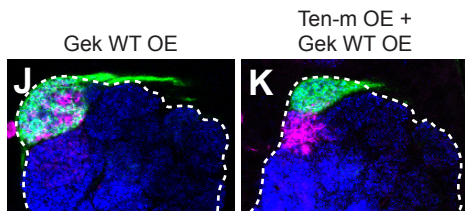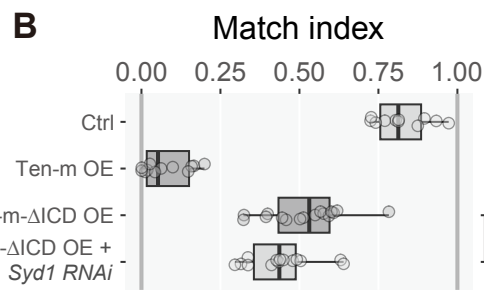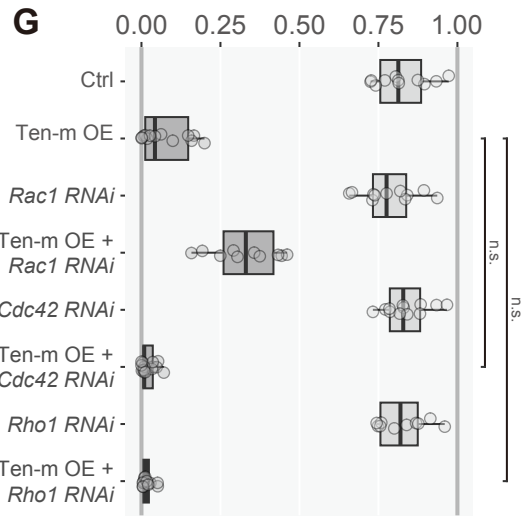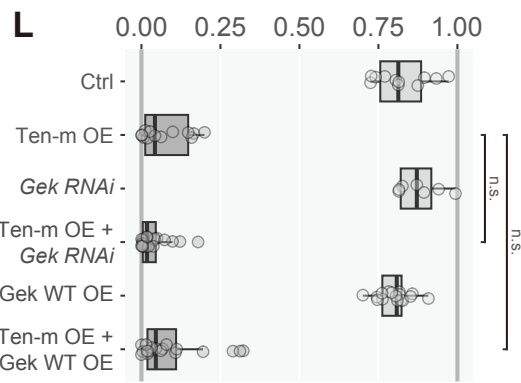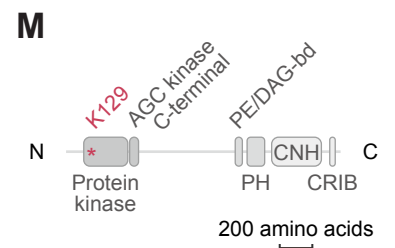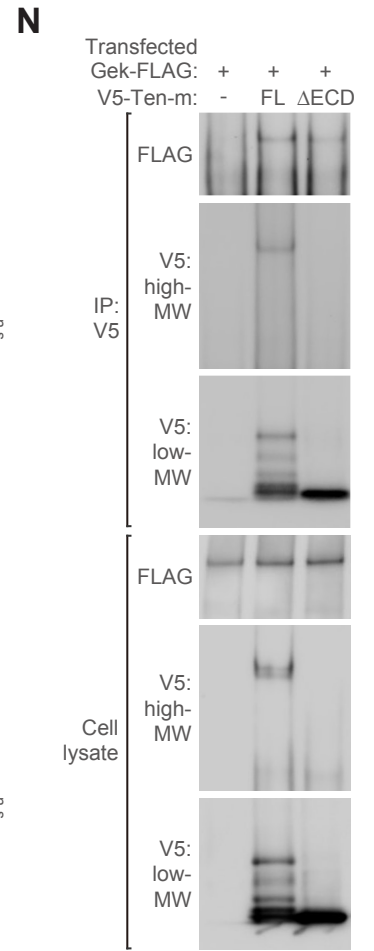

Supplement: 5 — Figure S4. Genetic interactions of Ten-m with Syd1, Cdc42, Rho1, and Gek in ORNs, related to Figures 3 and 4. (A) Representative confocal images of DA1-PN dendrites (magenta) and DA1-ORN axons (green) of Ten-m-ΔICD overexpression with Syd1-RNAi. (B) Match index of (A), which also includes Ten-m-ΔICD overexpression alone, as well as control and Ten-m overexpression data (from Figure 1M) for comparison. (C–G) Representative confocal images of DA1-PN dendrites (magenta) and DA1-ORN axons (green) of Cdc42-RNAi (C), Ten-m overexpression with Cdc42-RNAi (D), Rho1-RNAi (E), and Ten-m overexpression with Rho1-RNAi (F). Match indices are quantified in (G), which also includes Rac1-RNAi (same data as in Figure 3S) for comparison as well as the control and Ten-m overexpression data from Figure 3I. (H–L) Representative confocal images of DA1-PN dendrites (magenta) and DA1-ORN axons (green) of Gek-RNAi (H), Ten-m overexpression with Gek-RNAi (I), Gek overexpression (J), and Ten-m and Gek co-overexpression (K). Match indices are quantified in (L), which also includes the control and Ten-m overexpression data from Figure 3I. (M) Protein domain organization of Gek. The asterisk (*) marks the lysine in the protein kinase domain essential for its catalytic activity. (N) Co-immunoprecipitation of V5-tagged Ten-m and FLAG-tagged Gek proteins from co-transfected S2 cells. MW, molecular weight. D, dorsal; L, lateral. Dashed white circle, antennal lobe. BRP, Bruchpilot, an active zone marker used for general neuropil staining. Mann-Whitney U test was used for the comparison (B). Kruskal-Wallis test with Bonferroni post-hoc correction for multiple comparisons was used in (G, L). [file NIHMS2005581-supplement-5.pdf]

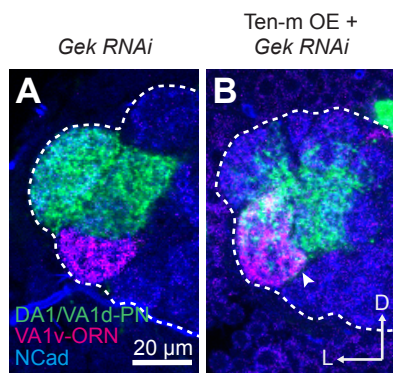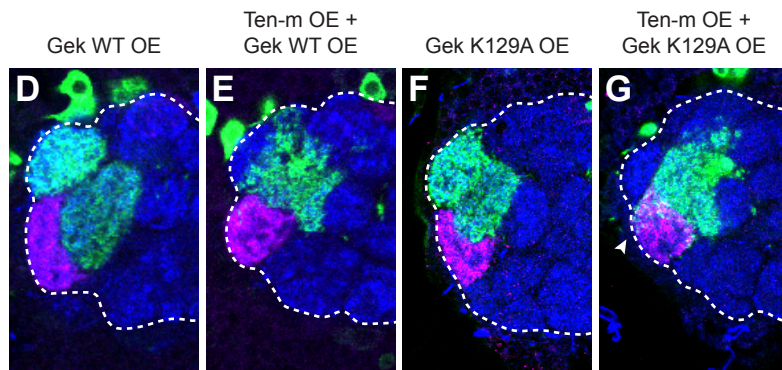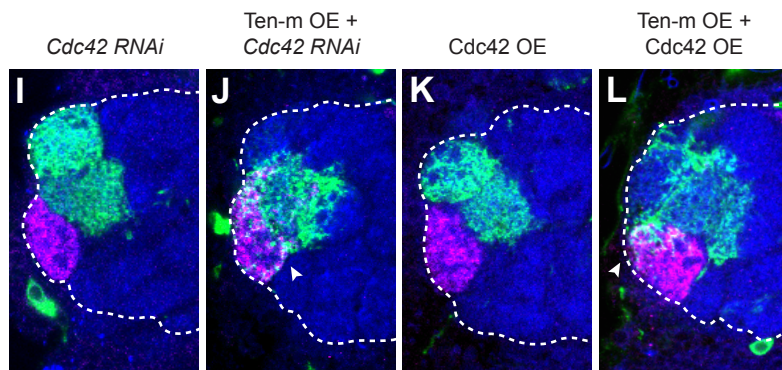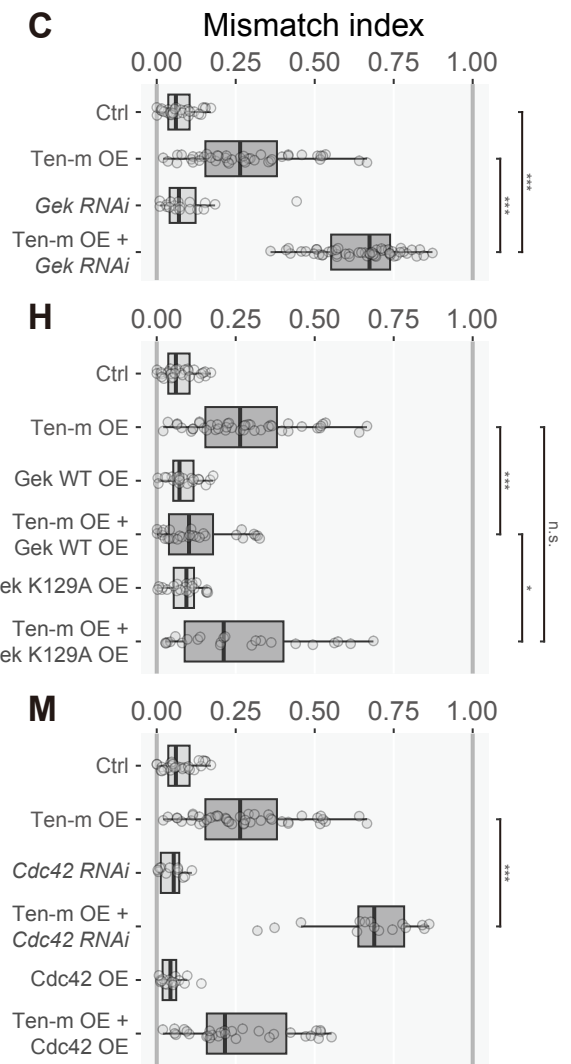

Supplement: 6 — Figure S5. Genetic interactions of Ten-m with Gek and Cdc42 in PNs, related to Figure 4. (A–C) Representative confocal images of VA1v-ORN axons (magenta) and Mz19-PN dendrites (green) of Gek-RNAi (A) and Ten-m overexpression with Gek-RNAi (B). Mismatching indices are quantified in (C), which also includes the control and Ten-m overexpression data from Figure 4G. (D–H) Representative confocal images of VA1v-ORN axons (magenta) and Mz19-PN dendrites (green) of Gek overexpression (D), Gek and Ten-m co-overexpression (E), Gek-K129A protein kinase-domain mutation overexpression (F), and Gek-K129A and Ten-m co-overexpression (G). Mismatch indices are quantified in (H), which also includes the control and Ten-m overexpression data from Figure 4G. (I–M) Representative confocal images of VA1v-ORN axons (magenta) and Mz19-PN dendrites (green) of Cdc42-RNAi (I), Ten-m overexpression with Cdc42-RNAi (J), Cdc42 overexpression (K), and Cdc42 and Ten-m co-overexpression (L). Mismatching indices are quantified in (M), which also includes the control and Ten-m overexpression data from Figure 4G. D, dorsal; L, lateral. Dashed white circle, antennal lobe. NCad, N-cadherin, a general neuropil marker. Arrowheads indicate overlap regions. Kruskal-Wallis test with Bonferroni post-hoc correction for multiple comparisons was used in (C), (H), and (M). [file NIHMS2005581-supplement-6.pdf]

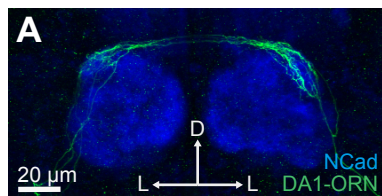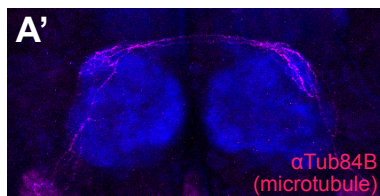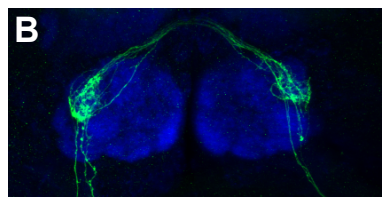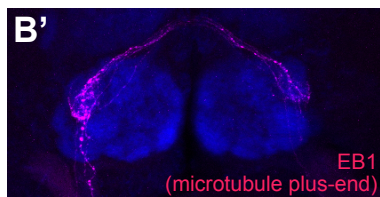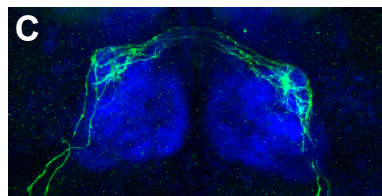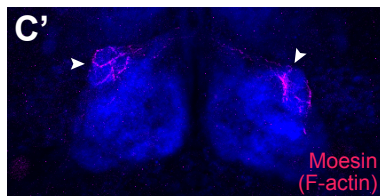

Supplement: 8 — Figure S7. Localizations of cytoskeleton markers in sparsely labeled developing ORN axons, related to Figure 7. Representative maximum Z-projection images of sparse DA1-ORN axons with microtubule marker Halo-alphaTub84B (A and A’), microtubule plus-end marker Halo-EB1 (B and B’), or F-actin marker Halo-Moesin (C and C’). Arrowheads indicate signal peaks of differential distribution of the F-actin marker. D, dorsal; L, lateral. NCad, N-cadherin, a general neuropil marker. [file NIHMS2005581-supplement-8.pdf]
