# Supplementary material for: Molecular and Cellular Mechanisms of Teneurin Signaling in Synaptic Partner Matching
Source: Cell. Author manuscript; Available in PMC 2025 Feb 18. (PMC11833509; doi:10.1016/j.cell.2024.06.022)
Supplement: 7 — Figure S6. The sparse driver strategy and single-axon analyses, related to Figure 6. (A) The sparse driver strategy incorporates hsFLP (FLP recombinase driven by a heat-shock promoter), heat shock, and mutant FRT (FRT10) sites, where the A→T mutation (red) reduces recombination efficiency by 10-fold. Following recombination, the in-frame peptide derived from FRT10 and T2A sequences is excised during the translation of the activation domain (AD). (B) Protocol for activating the sparse driver in a single DA1-ORN. (C, D) Representative maximum Z-projection images of a single DA1-ORN at Stage 2 (C) and Stage 3 (D). (E–G) 3D trace Z-projections of the DA1-ORN axons of control at Stage 1 (E), Stage 2 (F), and Stage 3 (G). (H–J) 3D trace Z-projections of the DA1-ORN axons of Ten-m overexpression at Stage 1 (H), Stage 2 (I), and Stage 3 (J). (K–M) 3D trace Z-projections of the DA1-ORN axons of Ten-m overexpression with Rac1-RNAi at Stage 1 (K), Stage 2 (L), and Stage 3 (M). D, dorsal; L, lateral. Orange square, axon entry point. Dark green, stem axon. Light green, axon branches. Yellow dot, primary branch point. [file NIHMS2005581-supplement-7.pdf]

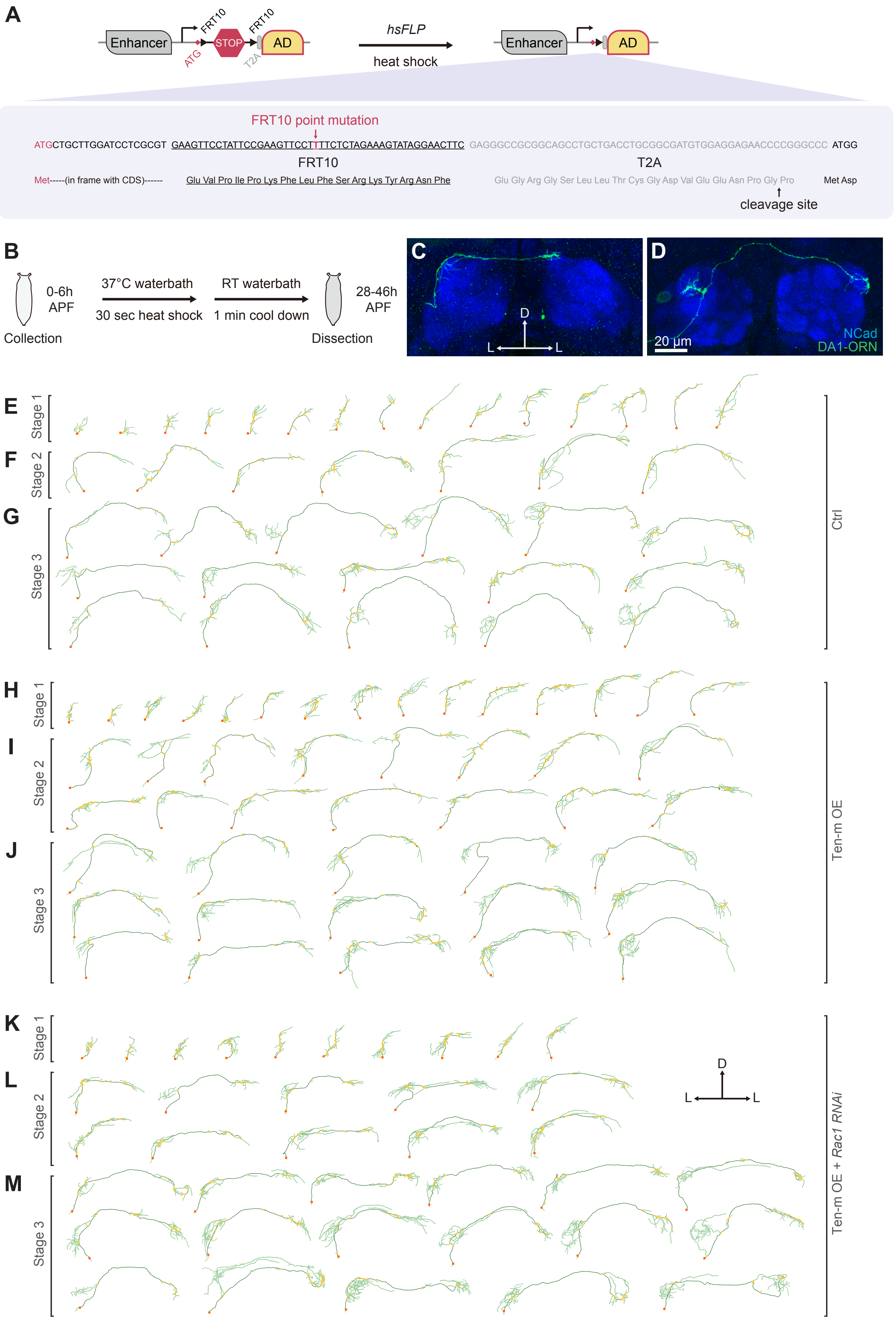
A
Enhancer
FRT10
FRT10
ATG
STOP
T2A
AD
hsFLP
heat shock
Enhancer
AD
FRT10 point mutation
ATGCTGCTTGGATCCTCGCGT GAAGTTCCTATTCCGAAGTTCCTTTTCTCTAGAAAGTATAGGAACTTC GAGGGCCGCGGCAGCCTGCTGACCTGCGGCGATGTGGAGGAGAACCCCGGGCCC ATGG
FRT10
T2A
Met-----(in frame with CDS)------ Glu Val Pro Ile Pro Lys Phe Leu Phe Ser Arg Lys Tyr Arg Asn Phe Glu Gly Arg Gly Ser Leu Leu Thr Cys Gly Asp Val Glu Glu Asn Pro Gly Pro Met Asp
cleavage site
B
0-6h APF
Collection
37°C waterbath
30 sec heat shock
RT waterbath
1 min cool down
28-46h APF
Dissection
C
D
L
L
D
20 μm
NCad
DA1-ORN
E
Stage 1
F
Stage 2
G
Stage 3
Ctrl
H
Stage 1
I
Stage 2
J
Stage 3
Ten-m OE
K
Stage 1
L
Stage 2
M
Stage 3
D
L
L
Ten-m OE + Rac1 RNAi
